# Prediction of Australian wheat genotype by environment interactions and mega-environments

**DOI:** 10.1007/s00122-025-05023-6

**Published:** 2025-09-04

**Authors:** Nick S. Fradgley, Guillermo S. Gerard, Velu Govindan, Julie M. Nicol, Amit Singh, Wuletaw Tadesse, Alexander B. Zwart, Richard Trethowan, Ben Trevaskis, Alex Whan, Jessica Hyles

**Affiliations:** 1 CSIRO Agriculture and Food, GPO Box 1700, Canberra, ACT 2601 Australia; 2 International Maize and Wheat Improvement Center (CIMMYT), KM 45 Carretera Mexico-Veracruz, 56237 Texcoco, Mexico; 3 The Plant Breeding Institute, University of Sydney, 107 Cobbity Road, Cobbity, NSW 2750 Australia; 4 International Center for Agricultural Research in the Dry Areas (ICARDA), PO Box 6299, Rabat, Morocco

## Abstract

**Key message:**

L**atent environmental effects of genotype by environment interactions could be predicted from observed environmental covariates. Predictions into the wider target population of environments revealed greater insights.**

**Abstract:**

Wheat is grown across a diverse range of environments in Australia with contrasting environmental constraints. Targeted breeding to optimise genotypes in target environments is hindered by large and ubiquitous genotype by environment interactions (GEI). Common GEI in multi-environment trial experiments, which sample the target population of environments, can be efficiently modelled using latent environmental effects from factor analytic mixed models. However, generalised prediction into the full target population of environments is difficult without a clear link to observed environmental covariates (ECs) that are defined from high-resolution weather and soil data. Here, we used a large wheat multi-environment trial dataset and demonstrated that latent environmental effects can be associated with and predicted from observed ECs. We found GEI-based environment classes could be defined by combinations of key ECs. Prediction of main and latent effects in a wider set of environments covering the full TPE across the Australian grain belt over 13 years revealed the complex trends of environmental effects and GEI over regional scales demonstrating high year-to-year variability. Regional environment types often shifted year-to-year. Cross-validation of forward genomic prediction into untested year environments demonstrated that increased accuracy is possible if estimated genetic effects are also accurate and ECs of new environments are known. These findings may guide Australian wheat breeders to better target specifically adapted material to mega-environments defined by static GEI while also considering broad adaptability and non-static GEI resulting from year-to-year variability.

**Supplementary Information:**

The online version contains supplementary material available at 10.1007/s00122-025-05023-6.

## Introduction

Australia is a major wheat producing nation. Between 2010 and 2022, Australia contributed between 5.1 and 15.4% of the 145.7 to 200 million tonnes of global exports (FAO 2024). Along with improved agronomy and crop management, significant genetic gains achieved by plant breeders have increased wheat yields in key Australian growing regions (Kirkegaard 2019; Sadras Lawson 2011), but production environments are highly variable across locations and seasons. Abiotic factors such as water limitation and high temperature stress (Flohr et al. 2017) combined with biotic factors, including soil-borne pathogens (Thompson et al. 1999) limit yields, and extensive genotype by environment interactions (GEI) limit breeders' selections across multiple environments (Basford and Cooper 1998; Cullis et al. 2000). Increased temperatures due to changing climates are likely to reduce the potential of widely adapted varieties and reduce selection efficiency, hence requiring greater diversity of adapted breeding materials (Xiong et al. 2024).

Wheat breeders in Australia work with a relatively refined gene pool and a few major varieties often dominate the acreage and market share (Brennan and Fox 1998; Cockram 2024), which is in contrast to other major wheat producing nations such as the US (Chai et al. 2022). The International Maize and Wheat Improvement Centre (CIMMYT) and the International Centre for Agricultural Research in the Dry Areas (ICARDA) are large international wheat breeding programmes that collaborate with Australian wheat breeders and researchers to introduce and evaluate new sources of yield potential and adaptation to biotic and abiotic stresses as part of the CIMMYT Australia ICARDA Germplasm Evaluation (CAIGE) programme (Trethowan et al. 2024).

Unpredictability of GEI is a long-standing problem that has limited targeted breeding for specific environments as well as resilience to year-to-year variability. Many statistical modelling approaches have been developed to understand and predict GEI in multi-environment trial (MET) experiments (Crossa et al. 2024; Malosetti et al. 2016). Whereas early methods simply regressed genotype performance against an environmental index defined by the environment mean yields (Finlay and Wilkinson 1963; Yates and Cochran 1938), more recent approaches have sought to replace this environmental index with biologically meaningful observed environmental covariates (ECs) so that prediction into untested environments is possible.

As an analogy to genotyping, methods for ‘envirotyping’ derive large numbers of ECs from high-resolution weather data to characterise crop growing environments (Costa-Neto et al. 2021; Resende et al. 2021; Xu 2016), and are often informed by mechanistic crop growth models (Cooper et al. 2020; Zhang et al. 2012). Factorial regression models can incorporate genotype-specific responses to individual ECs (Boer et al. 2007; Piepho et al. 1998), but are problematic to fit when more than a few ECs need to be used. Rogers and Holland (2022) used principal component dimension reduction of large EC matrices, while multiple ECs can be linearly combined to define multiple synthetic covariates for GEI terms in similar mixed model frameworks (Piepho 2022; Piepho and Blancon 2023; Tadese et al. 2024). In a contrasting approach, Jarquín et al. (2014) developed reaction norm models that used environmental relationship matrix (ERM) kernels, defined by many ECs, to efficiently model similarity among environments so that GEI was modelled as a single term by multiplication of genomic and environmental relationship matrices. These reaction norm models generally assumed equal weighting among all ECs, but using partial least squared regression as a preliminary step to learn environment-phenotype association was shown to increase predictive ability (Costa-Neto et al. 2023), while Rincent et al. (2019) developed methods to determine the optimum subset of ECs needed to calculate the ERM for main effects and GEI terms separately.

Factor analytic models approximate common environment-specific genetic effects to small numbers of latent environmental factors and allow efficient analysis of highly unbalanced or sparse METs (Burgueño et al. 2008; Piepho and Williams 2024; Smith et al. 2001; Smith and Cullis 2018). Latent factor loadings from factor analytic models can then be used to define interaction classes of environments (iClasses) with minimal genotype cross-over interactions (Smith et al. 2021). In comparison to traditional methods of visualising biplots of single vector decompositions of two-way tables of genotype by environment means (Kempton 1984), and assigning ‘which one where’ mega-environment groups of environments with the same single best genotype (Yan et al. 2000), the iClass method of environment clustering is advantages in that it is flexible to highly unbalanced MET datasets, the factor loadings are identified in a single-stage model rather than genotype means, combines more than two principal component loadings and separates out common and specific GE effects. The genotype score response slopes against each latent factor loading can then be used to identify adapted and stable genotypes (Smith et al. 2021). Smith et al. (2023) further extended this approach to separately model additive and non-additive genetic variance for application in predictive breeding for untested chickpea genotypes. Tolhurst et al. (2022) integrated ECs into factor analytic models using cotton breeding trial data, while other authors have recently investigated correlations between ECs and factor analytic derived latent factors to characterise environmental drivers of GEI and define parameters of target environment types in several crop and animal breeding species (Bakare et al. 2022; Callister et al. 2024; Fairlie et al. 2024; Oliveira et al. 2020; Rogers and Holland 2022; Sae-Lim et al. 2014). Two-stage multivariate prediction of latent factor loadings using ECs in partial least squares regression models was shown to improve predictive ability of tested rice and soybean genotypes in new environments (Araújo et al. 2024).

Methods and models to predict GEI and characterise environment types using ECs have proven useful for predicting into new environments. However, this assumes that ECs for future environments are already known, so application of these approaches is only possible for expected static GEI effects, which are consistent over years at particular locations, rather than due to non-static year-to-year GEI variability (Cullis et al. 2000). For breeders to develop genotypes that are specifically adapted to particular mega-environment regions that exhibit repeatable GEI (Yan et al. 2023), and that are broadly adaptable to year-to-year variability within mega-environments, large numbers of environments representing the target population of environments across space and time should be considered. Here, we use a large MET dataset of CAIGE bread wheat genotypes tested over 13 years in Australian growing environments to: (i) define latent environmental factors and iClass environment types; (ii) demonstrate that latent environmental effects can be related to, and predicted from, observed ECs; (iii) predict out to a large number of extended untested environments covering the Australian grain belt; (iv) evaluate if this framework enables more accurate genomic prediction of new genotypes in new year environments; and (v) evaluate the adaptation patterns of new breeding material introduced through the CAIGE programme.

## Methods

### Multi-environment trial data

**Fig. 1:**
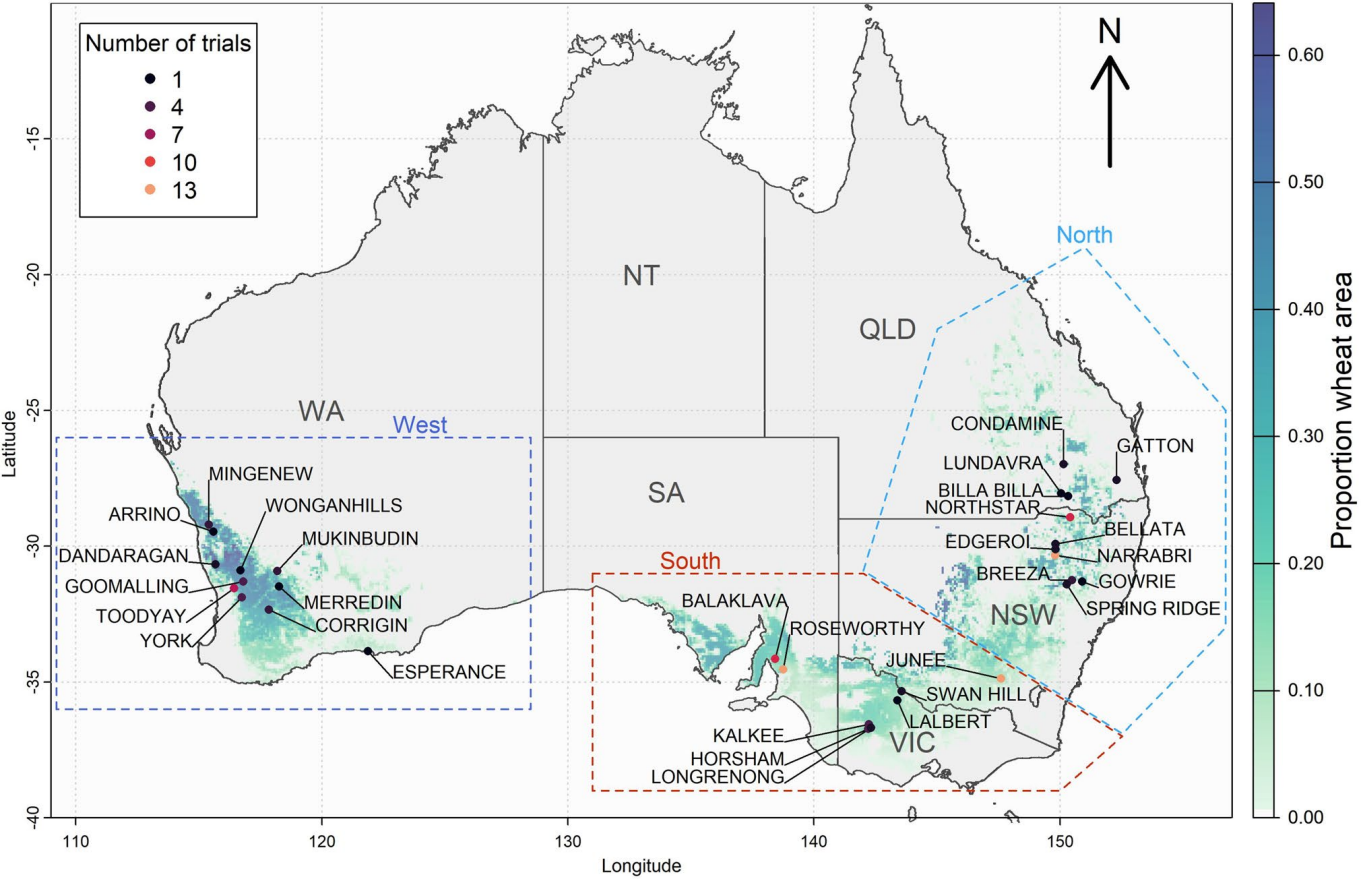
Map of CAIGE bread wheat multi-environment trial locations across three major wheat growing regions and the main Australian wheat growing areas in 2020 according to data from Monfreda et al. (2008)

A bread wheat MET dataset as part of the CAIGE project (Trethowan et al. 2024) included a total 34,293 trial plot observations of wheat grain yield for 3,355 genotypes (varieties, cultivars and breeding lines) tested across 114 trial environments (location by year combinations). This broadly covered the major Australian wheat growing regions. For the purposes of this study, we consider Basford and Cooper (1998) descriptions for trial environments in Western Australia (WA) as the western growing region, environments in South Australia (SA), Victoria (VIC) and southern New South Wales (NSW) as the southern growing region, and environments in northern NSW and Queensland (QLD) as the northern growing region (Fig. 1). Trial years spanned 2011 to 2023 with between three and 14 trials per year, and between 147 and 697 genotypes tested each year (Supplementary Table 1). Grain yield was the primary trait of interest but measures of days to heading and plant height were also obtained for a subset of key testing locations each year. Over 70% of trials were sown in May but sowing dates ranged from between the 17th of April to the 17th of August.

### Genetic material

A total of 1,691 and 1,546 genotypes from the CIMMYT and ICARDA breeding programmes, respectively, were tested across all years. Different cohorts of breeding lines from each of CIMMYT and ICARDA were tested each year (Supplementary Table 1). Genotypes were partially replicated across trial locations each year and each trial environment included between 114 and 475 entries. Very few breeding lines were tested in more than one trial year, but a set of 22 commercial varieties were tested across multiple years as check genotypes (Supplementary Table 2). On average, 188.5 (min = 12; max = 473) genotypes were in common between trials in the same year, but only two to 21 check entries were in common between trials in different years. The trial at Spring Ridge in 2020 was an exception to this where 227 entries from the 2019 trials cohort were included. An efficient sparse MET design with partial replication (Cullis et al. 2018, 2006) was used for each trial year. Across all trials, a median of 70.3% (min = 0%; max = 93.4%) of entries was single replicates while a median of 28.5% (min = 0%; max = 99.4%) of entries was replicated twice in each trial. The maximum number of replicates for any line in any single trial was between two (32.5% of trials) and eight (only at Narrabri in 2011).

### Genotypic analysis

Genotypic data were available for 2,058 (61.3%) of the genotypes tested in the MET. These included data from two genotyping platforms. Firstly, 6,530 markers for 1,432 CIMMYT breeding lines were derived from genotype by sequencing data, using methods described by Poland et al. (2012). Secondly, 20,512 single-nucleotide polymorphism (SNP) markers for 1,042 CIMMYT and ICARDA breeding lines and control varieties were genotyped using the Infinium Wheat Barley 40 K SNP array (Keeble-Gagnère et al. 2021). There were 416 lines genotyped with both marker platforms, and missing marker data were imputed based on linkage disequilibrium for all genotypes using a custom algorithm (R code for which is provided in Fradgley 2025b). Briefly, for each marker a subset of up to 500 markers with the greatest correlation to the focal marker and with differences in minor allele frequency < 0.1 were used to predict the categorical allele calls of the focal marker using random forest models via the ranger version 0.16.0 package (Wright and Ziegler 2017) in R version 4.4.0 (R Core Team 2023). This method did not require genetic or physical map marker positions. Only markers that could be predicted from a random forest model having out-of-bag prediction error < 0.2 were imputed. Accuracy of marker imputation was determined by cross-validation within the subset of lines genotyped on both platforms. 95.2% of marker calls were correctly imputed when randomly masking 10% of marker calls. Cross platform imputation accuracy of randomly masking all marker data from one platform for 10% of lines demonstrated average per marker imputation accuracy of 85.4% and 81.1% for markers on the Infinium Wheat Barley 40 K SNP and genotype by sequencing platforms, respectively. After imputation, markers with > 5% missing data, < 0.025 minor allele frequency or heterozygous call frequency > 0.1 were removed. The combined markers were also pruned to remove redundancy and bias between closely linked markers using a custom algorithm (R code for which is provided in  Fradgley  (2025b). Each marker was sequentially evaluated, and all other linked markers (defined as having correlation r < − 0.8 or r > 0.8) were removed. This resulted in *m* = 7,381 imputed and pruned markers for *n* = 2,058 genotypes. The *n* × *n* genomic relationship matrix (GRM) between all pairs of genotypes was then calculated following Endelman and Jannink (2012) using the A.mat function using the rrBLUP package in R as $\text{GRM}=\frac{{\varvec{M}}{\varvec{M}}\boldsymbol{^{\prime}}}{c}$, where ${{\varvec{M}}}_{ik}={{\varvec{X}}}_{ik}+1-2{p}_{s}$, ${\varvec{X}}$ is the *n* × *m* marker matrix, ${p}_{s}$ is the allele frequency at marker *s,* and $c=2{\sum }_{s}{p}_{s}(1-{p}_{s})$. A principal coordinate analysis plot of the GRM is shown in Supplementary Fig. 1.

Pedigree and selection history information associated with the lines and check varieties was also used to calculate the coefficient of parentage (COP) matrix among all pairs of 3,405 lines. Pedigrees formatted as text strings in Purdy format (Purdy et al. 1968) were converted to a three-column format with ID, P1 (parent 1) and P2 (parent 2) as column names using a custom script (R code for which is provided in Fradgley 2025b). Seven generations of inbreeding before crossing were assumed for each individual genotype except where a back-cross was specified by ‘*’ in the Purdy notation, where it was assumed that the back-cross was made to an F1 generation plant without inbreeding. Where available, selection histories for breeding lines were also used to determine partially shared inbreeding of lines within families from the same cross. The full pedigree in three-column format including intermediate generations of inbreeding was then used to calculate the COP matrix using the kinship function from the kinship2 version 1.9.6.1 package in R (Sinnwell et al. 2014).

### Environmental characterisation

Environmental data were collected from multiple sources to characteris Fe each trial environment. Daily weather records were accessed from the ‘SILO Patched Point Dataset—Australian climate data from 1889 to yesterday’ public database as described by Jeffrey et al. (2001). Weather variables included total daily rainfall (mm), daily minimum temperature (${T}_{min}$; °C), daily maximum temperature (${T}_{max}$; °C), total daily solar radiation (*SR*; MJ m^−1^) and daily vapour pressure deficit (*VPD*; hPa). Daily photothermal quotient ($\text{PQ}$; MJ m^−2^ day^−1^ °C^−1^) was also calculated as the ratio of photosynthetically active radiation to mean temperature as described by Dreccer et al. (2018), including the conversion of solar radiation to photosynthetically active radiation as applied by Pinker and Laszlo (1992), using the formula $\text{PQ}=\frac{\text{SR} \times 0.47}{({T}_{min}+{T}_{max})/2}$. The day lengths (h) were derived using each trial location latitude and the daylength function as part of the ChillR version 0.75 package in R (Luedeling et al. 2023).

As described by Heslot et al. (2014), daily weather data were processed to define biologically meaningful environmental stress covariates within estimated crop development stages. Similarly to Cane et al. (2013), key crop development stages were estimated based on cumulative crop thermal time ($TT$) growing degree days calculated for each day from trial sowing date with base, optimum and maximum cardinal temperature thresholds of 0, 26 and 34 °C, respectively, as outlined in the Agricultural Production Systems sIMulator (APSIM) wheat crop growth model (McCown et al. 1996). Where dates of sowing were not available, the median value across all known trial environments was assumed. The daily mean temperature ($T$) was calculated as $T=\frac{{T}_{max}+{T}_{min}}{2}$, and thermal time ($TT$) for each day was then calculated as:

$$TT = \left\{ {\begin{array}{*{20}l} T \hfill & {0 < T \le 26} \hfill \\ {\frac{26}{8}\left( {34 - T} \right)} \hfill & {26 < T \le 34} \hfill \\ 0 \hfill & {T \le 0 or T > 34} \hfill \\ \end{array} } \right.$$

A modified version of crop growth stage estimates described in APSIM (McCown et al. 1996) was used. The crop emergence growth stage (*Emer*), corresponding to growth stage (GS) 10 (Zadoks et al. 1974) was estimated to occur 14 days after sowing if more than three mm of rain occurred in the seven days prior to sowing date or 14 days after the first day of rain after sowing (*Sow*). The end of juvenile growth stage (*Juv*; GS30) was estimated to occur when 500 cumulative *TT* after *Emer* was reached. Based on a subset of trial environments in which days to heading (*He*; GS55) was recorded, the cumulative *TT* after *Sow* that median ear emergence across all plots occurred was estimated as 1,374, 1,301 and 1,194 *TT* for environments in the North, South and West, respectively. Flowering (*Flw*; GS65) was estimated to occur when 250 cumulative *TT* after *He* was reached. Start of grain filling (Sgf; GS71) was estimated to occur when 250 cumulative *TT* after *Flw* was reached. End of grain filling (*Egf*; GS87) was estimated to occur when 250 cumulative *TT* after *Sgf* was reached. Finally, maturity (*Mat*; GS92) was estimated to occur when 400 cumulative *TT* after *Egf* was reached. Thus, dates of seven crop growth intervals including *Sow2Emer*, *Emer2Juv*, *Juv2He*, *He2Flw*, *Flw2Sgf*, *Sgf2Egf* and *Egf2mat* were defined for each trial environment and a summary of all ECs is provided in Supplementary Table 4.

Growth interval specific environmental covariates (ECs) were then calculated from daily weather conditions between consecutive estimated crop growth stages. These included; the average values of *T*, ${T}_{min}$ and ${T}_{max}$ (*Avtemp*, *Avmintemp* and *Avmaxtemp,* respectively), day lengths (*AveDL*), solar radiation (*AveSR*) and vapour pressure deficit (*AveVPD*), in addition to the total number of days (*Ndays*), the total rainfall (*TotRain*), the number of dry days with zero rain (*Ndd*), the number of frost days when ${T}_{min}<0$ °C (*Ndays* < *0*), the number of warm days when ${T}_{max}>26^\circ \text{C}$ (*Ndays* > *26*) and the number of hot days when ${T}_{max}>34^\circ \text{C}$ (*Ndays* > *34*) for each interval between consecutive growth stages. For example, *Avtemp_He2Flw* denotes the average of daily mean temperature ($T$) between the heading and flowering crop growth stages. Additionally, ECs over multiple intervals were calculated which included the total rain between *Sow* and *Flw* (*TotRain_Sow2Flw*), total rain between *Flw* and *Egf* (*TotRain_Flw2Egf*), prior stored soil moisture estimated as the total rain between the 1st of January and the date of sowing (*TotRain_priorSow*), the number of days between *Sow* and *Flw* (*Ndays_Sow2Flw*) and the number of days between *Flw* and *Egf* (*Ndays_Flw2Egf*). The number of frost days when ${T}_{min}<0$ °C within seven days of the estimated flowering day (*Mintemp* < *0_Flw*) was also calculated.

ECs also included soil characteristics for each trial location derived from the Soil and Landscapes Grid of Australia dataset (Grundy et al. 2015) using the SLGACloud version 1.0.1 package in R. Point data were extracted for the 90 m^2^ grid closest to latitude and longitude coordinates of each trial location. Soil attributes were taken from the national soil attributes map at 0–5, 5–15, 15–30, 30–60, 60–100 and 100–200 cm soil depth layers. These included: soil texture percentages of clay, silt and sand; available water capacity (%); bulk density of whole earth including coarse fragments (g cm^−1^); effective cation exchange capacity (meq 100 g^−1^); organic carbon (%); mineral associated, particulate and pyrogenic organic carbon (%); coarse fragments (%); pH of water; fraction of both total nitrogen and phosphorus (%), available phosphorus (mg kg^−1^); as well as the volumetric drained upper and lower limits (%). Depth of soil profile A and B horizons (m) was also extracted. ECs with zero variance across trial environments were removed leaving a total of 96 weather stress ECs and 90 soil characteristic ECs. Finally, the total of *w* = 186 weather and soil ECs were combined into an *p* × *w* matrix (***W***).

We also derived ECs in the same manner as described above for 369,161 extended environments in the Australian wheat belt, over 13 growing seasons from 2011 to 2023, using historical gridded weather data. The mean sowing date across all environments in the observed MET of the 24th of May was assumed for all extended environments. Locations were selected from a grid of 572,721 latitude and longitude coordinates covering Australia with 681 latitude values between -44 and -10, and 841 longitude values between 112 and 154. Of these, 28,397 locations were selected which had a fraction of wheat cropping area > 0.02 according to data from Monfreda et al. (2008) using the geodata version 0.6–2 package in R. Functions for deriving ECs are provided in the EC4MET R package (Fradgley and Whan 2025).

### Multi-environment trial analyses

Data from the multi-environment trials (MET) were analysed with linear mixed models fitted using the ASREML-R version 4.2.0.276 software package (Butler et al. 2017) in R. One-stage MET analyses including all data from individual trial plots across all environments with specific random and residual spatial covariance structures for each trial environment were considered superior in terms of statistical efficiency compared to two-stage models (Damesa et al. 2017; Gogel et al. 2018; Welham et al. 2010). To enable fitting of a one-stage model, we first progressively determined parsimonious model structures for each trial environment separately using single-trial and single-year models. Fitted parameters from these simpler models were used as average information algorithm parameter starting values for the one-stage MET model as detailed below. R code for MET analyses are provided in Fradgley (2025b).

#### Single-trial analyses

Following Smith et al. (2021), linear mixed models with genetic and non-genetic effects were fitted as ${\varvec{y}}={\varvec{X}}{\varvec{\tau}}+{{\varvec{Z}}}_{{\varvec{g}}}{{\varvec{u}}}_{{\varvec{g}}}+{{\varvec{Z}}}_{{\varvec{p}}}{{\varvec{u}}}_{{\varvec{p}}}+{\varvec{\varepsilon}}$, where ${\varvec{\tau}}$ is a vector of fixed effects with the associated design matrix ***X***; ${{\varvec{u}}}_{{\varvec{g}}}$ is the vector of random genetic effects associated with the design matrix ${{\varvec{Z}}}_{{\varvec{g}}}$; ${{\varvec{u}}}_{{\varvec{p}}}$ is the vector of random non-genetic peripheral effects associated with the design matrix ${{\varvec{Z}}}_{{\varvec{p}}}$ and ${\varvec{\varepsilon}}$ is the vector of residual errors. A total of 28 models were fitted for each trial, with genotype identity as a random factor effect (${{\varvec{u}}}_{{\varvec{g}}}$) and testing all combinations of trial row and column coordinates factor peripheral random effects, in addition to row and column independent (id), auto-regressive order 1 (ar1) and auto-regressive order 2 (ar2) residual structure non-genetic effects (${{\varvec{u}}}_{{\varvec{p}}}$) (Supplementary Table S3). For each trial, the model with the lowest Akaike information criterion (AIC) was selected as the best fitting model and the estimated random and residual effects parameters for these were recorded and recycled for use in multi-environment models. Model residually were visually assessed for normality and to identify outlier plot values.

Prediction error variance (PEV) of each estimated genetic effect per line was calculated as $\text{PEV}=$ SE^2^_,_ where SE denotes the standard error. Reliability (*r*) of each estimate was calculated as: $r=1-\text{PEV}/{\sigma }_{G}^{2}$, where ${\sigma }_{G}^{2}$ is the genetic variance. Seven environments with low mean reliability of all genotype estimates per environment (*r* < 0.3) were removed from further multi-environment analyses.

#### Single-year MET analyses

To determine suitable starting values for multi-year FA1 models, linear mixed models for all trials within each year were then fitted as detailed by Smith et al. (2023). Trial environment identity was included as a fixed main effect (${\varvec{\tau}}$) and genetic random effects using either the GRM or COP to model genetic covariances among genotypes (${{\varvec{G}}}_{{\varvec{n}}}$). Considering *p* as the number of environments and *n* the number of individuals with genotype data that are present in the GRM or that have pedigree data and are included in the COP, the total genetic variance ${\varvec{G}}$ is defined as ${\varvec{G}}={{\varvec{G}}}_{{\varvec{e}}}\otimes {{\varvec{G}}}_{{\varvec{n}}}$, where ${{\varvec{G}}}_{e}$ is the *p* × *p* matrix of genetic covariance between environments, ${{\varvec{G}}}_{{\varvec{n}}}$ is the *n* × *n* GRM or COP numerator matrix and $\otimes$ indicates the Kronecker product. The ${{\varvec{G}}}_{{\varvec{e}}}$ matrix was first structured for a baseline model having diagonal (DIAG) variance–covariance structure that assumes a specific genetic variance (${\sigma }^{2}$) at each of *e* environments but with independent genetic covariance (0) among all pairs of *p* environments:

$${{\varvec{G}}}_{{\varvec{e}}}= \left[\begin{array}{cccc}{\sigma }_{1}^{2}& 0& \cdots & 0\\ 0& {\sigma }_{2}^{2}& \cdots & 0\\ \vdots & \vdots & \ddots & \vdots \\ 0& 0& \cdots & {\sigma }_{p}^{2}\end{array}\right]$$

Then, reduced rank factor analytic order 1 (FA1) models that approximated heterogeneous genetic covariance among environments in ${{\varvec{G}}}_{{\varvec{e}}}$ using a single factor (*k* = 1) were fitted for all trials within each year. Reduced rank factor analytic (FA_*k*_) models combine both reduced rank and diagonal components as ${{\varvec{G}}}_{{\varvec{e}}}={\varvec{\Lambda}}{\varvec{D}}{{\varvec{\Lambda}}}^{\mathbf{^{\prime}}}+{\varvec{\Psi}}$, where ${\varvec{\Lambda}}$ is a matrix of *p* × *k* factor loadings, ${\varvec{D}}$ is a *k* × *k* matrix of factor score variances and ${\varvec{\Psi}}$ is the ${\sigma }_{1}^{2},{\sigma }_{2}^{2},\dots ,{\sigma }_{e}^{2}$ diagonal (non-identity) matrix of environment-specific genetic variances. Therefore, ${\varvec{\Lambda}}{\varvec{D}}{{\varvec{\Lambda}}}^{\mathbf{^{\prime}}}$ accounts for common and repeatable genotype nested with environment effects (GE) occurring in more than one environment which can be approximated by the FA structure, while ${\varvec{\Psi}}$ accounts for environment-specific GE effects that are not repeatable across tested trial environments. Rather than fitting both additive and non-additive effects with separate FA structures as implemented by Smith et al. (2023) and Smith and Cullis (2018), we only fitted additive genetic effects considering the GRM or COP similarly to Tolhurst et al. (2022).

To aid convergence of the mixed model average information (AI) algorithm, REML starting values for trial specific random and residual covariance structure row and column effects (${{\varvec{u}}}_{{\varvec{p}}}$) for single-year DIAG models were recycled from the single-trial analyses best fitting models. Starting values for single-year FA1 models specific genetic variances (${\varvec{\Psi}}$), as well as non-genetic random effects variance parameters (${{\varvec{u}}}_{{\varvec{p}}}$) were recycled from fitted parameters from converged single-year DIAG models. Auto-regressive row and column covariance effects for environments that prevented single-year models from converging were adjusted to independent ‘id(ROW):id(COL)’ for further analyses.

#### Full multi-year MET analyses

Full one-stage models were finally fitted using all plot data across all environments with high mean reliability (r > 0.3) from all trial years including either the GRM or COP as ${{\varvec{G}}}_{{\varvec{n}}}$. Note that at this stage, the dimension size (*n*) of the ${{\varvec{G}}}_{{\varvec{n}}}$ matrices were *n* = 2,058 and 3,405 for GRM and COP models, respectively. Like the single-year analyses described above, the one-stage DIAG model was first fitted using ${\varvec{\tau}}$ fixed environmental main effects and ${{\varvec{u}}}_{{\varvec{p}}}$ parameters starting values recycled from single-year DIAG models. Then reduced rank FA models with incrementally increased *k* factors were progressively fitted up to *k* = 3 when, as suggested by (Smith et al. 2015), more than 80% of the total variance was explained. For the one-stage FA1 model, ${\varvec{\tau}}$ and ${{\varvec{u}}}_{{\varvec{p}}}$ parameter starting values were recycled from the one-stage DIAG model and ${{\varvec{u}}}_{{\varvec{g}}}$ parameter starting values including ${\varvec{\Psi}}$ and the first factor loadings were recycled from respective single-year FA1 models. All possible ${\varvec{\tau}}$, ${{\varvec{u}}}_{{\varvec{g}}}$ (including *k* > 1 factor loadings) and ${{\varvec{u}}}_{{\varvec{p}}}$ starting values for subsequent FA2 and FA3 models were recycled from lower order (*k*-1) models.

As described by Smith et al. (2021), the estimated ${\varvec{\Lambda}}$ matrix of factor loadings and associated genotype scores were rotated to a principal component solution using the svd function in R. ${{\varvec{G}}}_{e}$ covariance matrices estimated from rotated ${\varvec{\Lambda}}$ could then be converted to genetic correlation matrices among all pairs of environments using the cov2cor function in R.

The percentages of variance explained by each *r* factor loading for each of *p* environments (${{\varvec{v}}}_{{{\varvec{r}}}_{{\varvec{e}}}}$) was calculated as ${{\varvec{v}}}_{{{\varvec{r}}}_{{\varvec{e}}}}=100 ({{\varvec{D}}}_{kk}{\text{diag}({\varvec{\Lambda}}}_{k}{{\varvec{\Lambda}}}_{k}^{\mathbf{^{\prime}}}))/\text{diag}({{\varvec{G}}}_{e})$, where ${{\varvec{D}}}_{kk}$ is the value of factor score variance for factor *k*, ${{\varvec{\Lambda}}}_{k}$ is the vector of environment factor loadings for factor *k* and $\text{diag}$(${{\varvec{G}}}_{e}$) is the diagonal vector component of the ${{\varvec{G}}}_{e}$ matrix. The total variance explained by the full model including all factors ($\overline{{\varvec{v}}}$) was calculated as $\overline{{\varvec{v}}}=100 \text{tr}({\varvec{\Lambda}}{\varvec{D}}{{\varvec{\Lambda}}}^{\mathbf{^{\prime}}})/\text{tr}({{\varvec{G}}}_{e})$, where $\text{tr}({{\varvec{G}}}_{e})$ computes the trace, i.e. the sum of the diagonal component of ${{\varvec{G}}}_{e}$.

Each environment was assigned to one of several defined interaction classes (iClasses) environment types. These iClasses minimise cross-over interactions from common GE effects within classes (considering that changes in rank ordering of genotype scores for each factor only occur when each factor loading equals zero) (Burgueño et al. 2008; Smith et al. 2021, 2023). This method uses the vector of *k* rotated factor loadings for each of *e* environments (${{\varvec{\Lambda}}}_{e}$) where positive loadings are assigned as ‘p’ and negative loadings are assigned to ‘n’. These vectors of characters are then concatenated to iClass strings of length *k* with 2^*k*^ possible iClasses. As proposed by Smith et al. (2023), iClass groups containing only one environment were merged with the iClass with the highest mean genetic correlation. Environment iClasses were then compared across geographic regions and years to illustrate spatial and temporal drivers of GEI.

Genotype scores ($\widetilde{f}$) from FA models describe the regression slopes responses of each genotype to each factor loading and are used to estimate environment-specific empirical best linear unbiased predictions (EBLUPs). As detailed by Smith and Cullis (2018), general overall performance (OP) across all iClasses for genotype *i* was defined as ${\text{OP}}_{i}= {\overline{\Lambda } }_{1}{\widetilde{f}}_{1i}$, where ${\overline{\Lambda } }_{1}$ is the average first factor loading across environments, and ${\widetilde{f}}_{1i}$ is the first factor genotype score for genotype *i*. Note that the OP score will not apply a minority of environments with negative first factor loadings. Similarly, the iClass overall performance (iClassOP) for each genotype *i* in each iClass $\omega$ was calculated as:

$${\text{OP}}_{\omega i}=\sum_{r=1}^{k}{\overline{\Lambda }}_{r\omega }{\widetilde{f}}_{ri}$$

where ${\overline{\Lambda }}_{r\omega }$ is the mean factor loadings for factor *r* of all environments in iClass $\omega$, and ${\widetilde{f}}_{ri}$ is the estimated genotype scores for genotype *i* and factor *r* (Smith et al. 2021; Smith and Cullis 2018). A genotype stability index based on the root mean squared error (RMSD) of higher order factor scores was also then calculated as:

$$\text{RMSD}=\sqrt{\frac{1}{p}\sum_{j=1}^{p}{{\widetilde{\in }}_{ij}}^{2}}$$

where ${\widetilde{\in }}_{ij}$ is the deviations of the linear regression line of common GE effects against first factor loadings for genotype *i* in environment *j*. Environment-specific EBLUPs integrating common GE effects could be calculated for any new environment where factor loadings (${\varvec{\Lambda}}$) can be predicted.

### Predictability of environment main effects and factor loadings

To increase interpretability and predictability of iClasses, we then investigated associations between both estimated environmental main effects and latent environmental factors against observed crop stress ECs. Pearson’s correlation coefficients were calculated between all combinations of *w* ECs and each of ${{\varvec{\Lambda}}}_{1:k}$ factor loadings vectors in addition to estimated environment main effects. One thousand permutations of randomly sampling each FA loadings vector were used to define 95% confidence interval thresholds for associations with ECs.

We then tested several approaches for predicting ${{\varvec{\Lambda}}}_{1:k}$ vectors of factor loadings and environmental main effects in a second-stage prediction model from *w* EC predictors. Note that this approach used fixed rather than random effect estimates of environmental main effects from the linear mixed model because they were used again to fit the second-stage model from which predictions were made to new environments. With more predictor ECs than environments (*w* > *p*), we tested multivariate prediction models including: random forest (RF) regression using ensembles of decision trees (Breiman 2001); a regularisation approach including the least absolute shrinkage and selection operator (LASSO; Tibshirani 1996); and partial least squares regression (PLS; Helland 1990). RF models were fitted using the randomForest version 4.7–1.1 package in R (Liaw and Weiner 2022) including default parameters of 500 trees, one-third of the variables randomly sampled at each split, and a maximum node size of five. Feature importance scores from full fitted models were also used to identify highly influential ECs. LASSO models were fitted using the glmnet version 4.1–8 package in R (Friedman et al. 2010) where optimum values of lambda for each model were selected based on tenfold cross-validation for 100 lambda values between 1 × 10^–8^ and 0.04.

Predictive ability of these models was assessed for a leave-one-environment-out cross-validation scheme as the Pearson’s correlation coefficients and the root mean squared errors (RMSE), calculated as $\text{RMSE}=\sqrt{\frac{\sum {\left({{\varvec{y}}}_{{\varvec{o}}{\varvec{b}}{\varvec{s}}}-{{\varvec{y}}}_{{\varvec{p}}{\varvec{r}}{\varvec{e}}{\varvec{d}}}\right)}^{2}}{p}}$, where ${{\varvec{y}}}_{{\varvec{o}}{\varvec{b}}{\varvec{s}}}$ and ${{\varvec{y}}}_{{\varvec{p}}{\varvec{r}}{\varvec{e}}{\varvec{d}}}$ are the observed and predicted values of each FA loadings and environmental main fixed effects, respectively. To account for shrinkage in predictions of environment main effects, out-of-fold predictions were adjusted so that the mean and variance of predictions equalled those of observed values as ${{\varvec{y}}}_{\mathbf{a}\mathbf{d}\mathbf{j}}=\frac{{\varvec{y}}-\overline{y} }{\sqrt{\sum ({{\varvec{y}}}^{2})/e-1}} \times \sqrt{\sum ({{{\varvec{y}}}_{\mathbf{o}\mathbf{b}\mathbf{s}}}^{2})/e-1}+{\overline{y} }_{\text{obs}}$, where ***y*** is the vector of predictions, $\overline{y }$ is the mean of the predictions, ${{\varvec{y}}}_{{\varvec{o}}{\varvec{b}}{\varvec{s}}}$ is the vector of observed values, ${\overline{y} }_{obs}$ is the mean of the observed values and *e* is the number of environments.

After predictive ability of models for environmental main effects and factor loadings influencing GE was validated, we then made predictions into the larger set of 369,161 extended environments as described above. To ensure realistic variance for the yield predictions, bias and shrinkage in predictions of environmental main effects were adjusted using intercept and slope linear regression coefficients from linear models fitted between observed and out-of-fold predicted main effect values as described above.

### Genomic prediction in untested environments

To validate the approach of prediction of factor analytic mixed model environmental main effects and factor loadings for untested environments, forward cross-validation of genotype performance prediction was applied. The cross-validation involved four rounds of forward prediction including trial environments between 2020 to 2023, 2021 to 2023, 2022 to 2023 and only 2023 as test sets of environments and using all environments from preceding years as training datasets to fit models. First, a genetic main effects only model (MM) was fitted as a baseline model which included only a single vector of ${{\varvec{u}}}_{{\varvec{g}}}$ genetic effect (not with covariance structures nested within environments) so that genetic effects were the same across all environments. Effects for peripheral (${{\varvec{u}}}_{{\varvec{p}}}$) effects were fitted with starting values from single-year models as described above. More complex models were then sequentially fitted from a DIAG_GRM_ model to an FA3_**GRM**_ model including lines present in the test years in the GRM, as detailed above. Genetic effects for each line from DIAG models were averaged across all environments in the training set of environments, and these averaged effects were used for predictions in all test environments. For all FA models, as also detailed above, random forest models were trained on the estimated factor loadings and environmental main effects from models fitted on the training set of environments with observed ECs as predictors. Factor loadings and environmental main effects for test set year environments were then predicted, and EGBLUPs for all genotypes in all test environments were calculated. Therefore, MM and DIAG models made no predictions of GE in the test environments, whereas FA models did predict environment-specific genotype performance.

Within-environment predictive ability of tested and untested genotypes for each test environment was calculated as the Pearson’s correlation and RMSE between predicted values and observed genetic effects estimated from single-trial analyses. Statistically significant differences between mean values of within trial environment predictive ability for the DIAG and each FA models were compared to the baseline MM model using paired two-tailed t-tests with the t.test function in R.

## Results

We analysed a large wheat MET dataset including 3,354 genotypes and 114 field trial environments to characterise genotype by environment interactions and associated latent environmental effects. Data from weather and soil environmental covariates could then be related to latent environmental effects to characterise GEI-based environment types (iClasses) for Australia.

### Model comparisons

By sequentially fitting single-trial, then single-year analysis models, a full one-stage model incorporating 32,472 yield plot observations could be fitted. Linear mixed models included reduced rank factor analytic (FA) approximation of heterogeneous genetic covariance structure among environments up to FA order *k* = 3 with either pedigree (COP) or genomic (GRM)-based relationship matrices. The COP models estimated more coefficients had lower values for AIC and BIC and greater log likelihood values relative to the GRM models (Table 1). This reflects the greater number of lines with pedigree information compared to those with genetic data and therefore greater capacity to fit larger COP models. The lowest AIC and BIC values across FA model orders differed for both COP and GRM models so similarly to Smith et al. (2015) and Tolhurst et al. (2022), we took a pragmatic approach considering the percentage of variance explained per environments. GRM models explained a greater proportion of the phenotypic variance than COP models. On average across all environments, 84.6% of the variance was accounted for per environment and more than two-thirds (69.0%) of environments had greater than 80% variance accounted for (Table 1). FA3_*GRM*_ was the only model to explain more than 80% of the total variance and so was chosen as the best fitting model and used to further define clusters of environments with similar GE. The variance in estimated coefficients of common GE effects that were approximated by the reduced rank FA structure was 0.148 and much larger than that of environment-specific genetic effects (0.007) which models non-repeatable GE. In fact, over half (51.8%) of the environments had near-zero (< 0.0001) specific genetic variance.

**Table 1:** Summary statistics of all fitted one-stage models for multi-year MET analyses

Model	Parameters	Coefficients	AIC	BIC	LL	% var
DIAG_*COP*_	522	364,436	− 15,704.57	− 11,321.47	8,374.29	–
FA1_*COP*_	742	729,515	− 20,244.21	− 14,937.47	10,754.11	53.53
FA2_*COP*_	852	732,804	− 21,285.98	− 15,062.99	11,383.49	71.68
FA3_*COP*_	962	736,093	− 21,641.58	− 14,512.74	11,669.79	76.54
DIAG_*GRM*_	522	227,926	− 12,609.19	− 8,226.09	6,826.59	–
FA1_*GRM*_	742	455,254	− 14,805.28	− 9,498.53	8,034.64	65.39
FA2_*GRM*_	852	457,302	− 15,117.69	− 8,895.70	8,299.84	76.58
FA3_*GRM*_	962	459,350	− 15,531.96	− 8,403.12	8,614.98	86.94

AIC is the Akaike Index Criterion, BIC is the Bayesian Index Criterion, LL is log likelihood and % var is the percentage variance explained

### Interaction classes in observed multi-environment trials

Estimates of environmental main effects in yield varied widely from 0.14 t ha^−1^ at Junee in 2019 to 6.97 t ha^−1^ at Balaklava in 2016. As expected, almost all (100 of 110) environments had positive loadings on the first factor which explained 50.0% of the total variance. This indicated that cross-over GEI was rare over the first factor while it mainly accounted for heterogeneous scaling of within-environment genetic variances across environments; higher yielding environments generally had greater genetic variance. Indeed, the correlation coefficient between the first factor loading and the estimated environmental main effects was 0.42. The second factor accounted for 26.3% of the GE variance with more balanced positive and negative loadings values (83 and 27 environments, respectively), representing the most important latent environmental effects influencing common cross-over GE. The second factor loadings were completely uncorrelated with environmental main effects (r = 0.01) suggesting that these environmental effects on yield are highly dependent on genotype with minimal overall yield impacts. Finally, the third factor accounted for 10.6% of the GE variance had 57 and 53 environments with positive and negative factor loadings, respectively, and was more closely correlated with environment main effects (r = − 0.40).

**Fig. 2:**
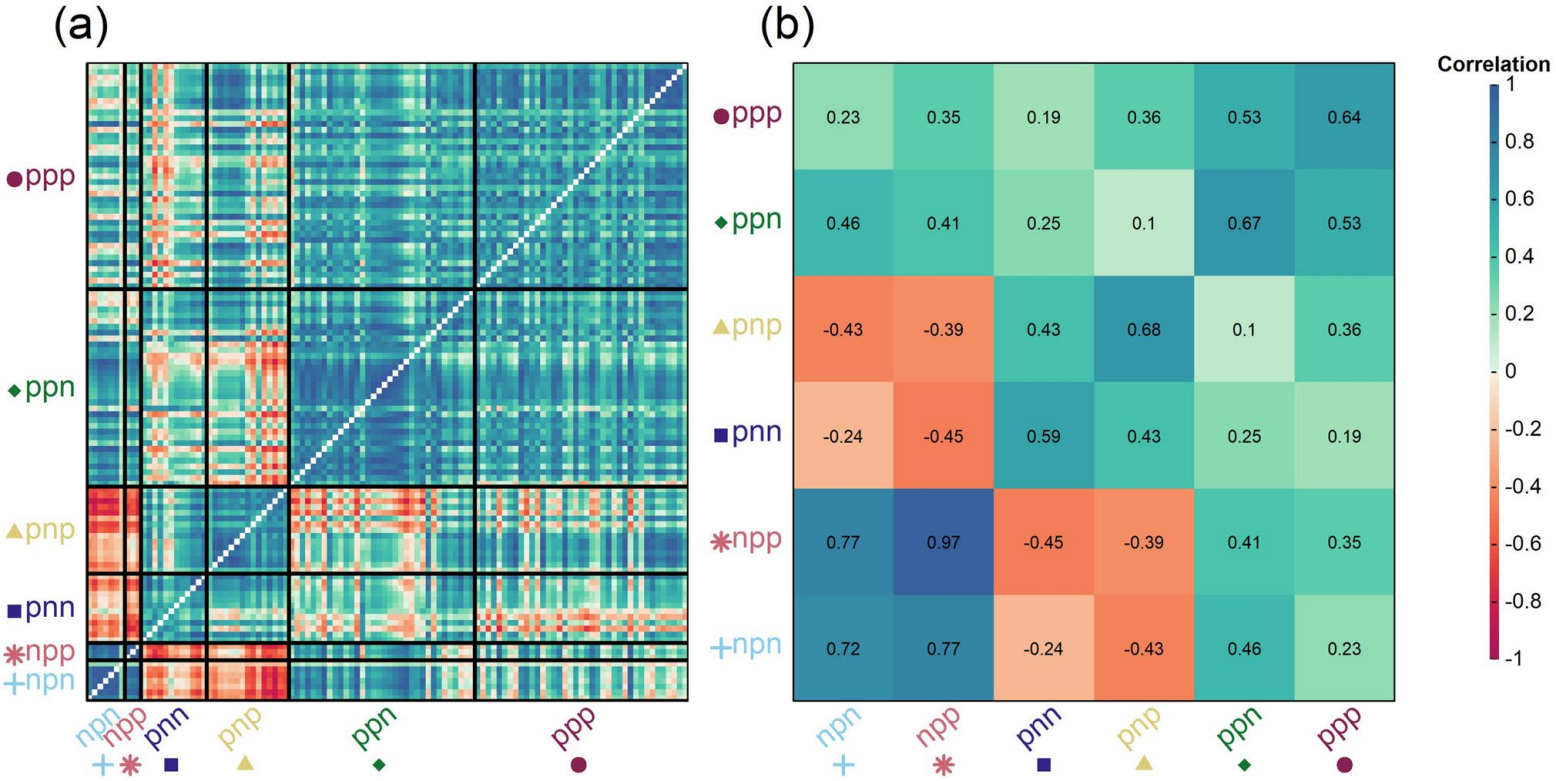
Heatmaps of genetic correlations among all pairs of environments ordered by iClasses (**a**); and mean values of genetic correlations among pairs of environments within (diagonal) and between (off-diagonal) the six iClass groups (**b**)

We used the iClass environment type clustering system to define groups of environments with the same sign (‘p’ = positive, ‘n’ = negative) of factor loadings, and minimal cross-over interactions (similar genotype ranking) within classes. The npp iClass was rarely identified, including only three environments whereas ppp was the most frequent, including 39 environments. Genetic correlations among all environments, estimated from the factor loadings and environment-specific genetic variances, ranged from − 0.90 to 0.99 with a mean of 0.40 across all pairs of environments (Fig. 2a). The two rarest iClasses (npp and npn) had small negative first factor loadings but still had relatively high genetic correlations with equivalent iClasses with positive first factor loadings (ppp and ppn, respectively). Genetic correlations among environments within iClasses groups were greater, ranging from 0.59 to 0.97 with a mean of 0.70, whereas genetic correlations among environments in different iClasses ranged from − 0.45 to 0.77 with a mean of only 0.17 across all pairs of iClasses (Fig. 2b). These results highlight the large GEI effects among the broad range of Australian wheat growing environment types. Of note, the ppn iClass was the second most frequently characterised, in 34 environments. The ppn iClass exhibited high within-class genetic correlations (ranging from 0.35 to 0.999 with a mean of 0.67), and so is an example of a frequently occurring class of environments with relatively consistent genetic effects and low GEI.

**Fig. 3:**
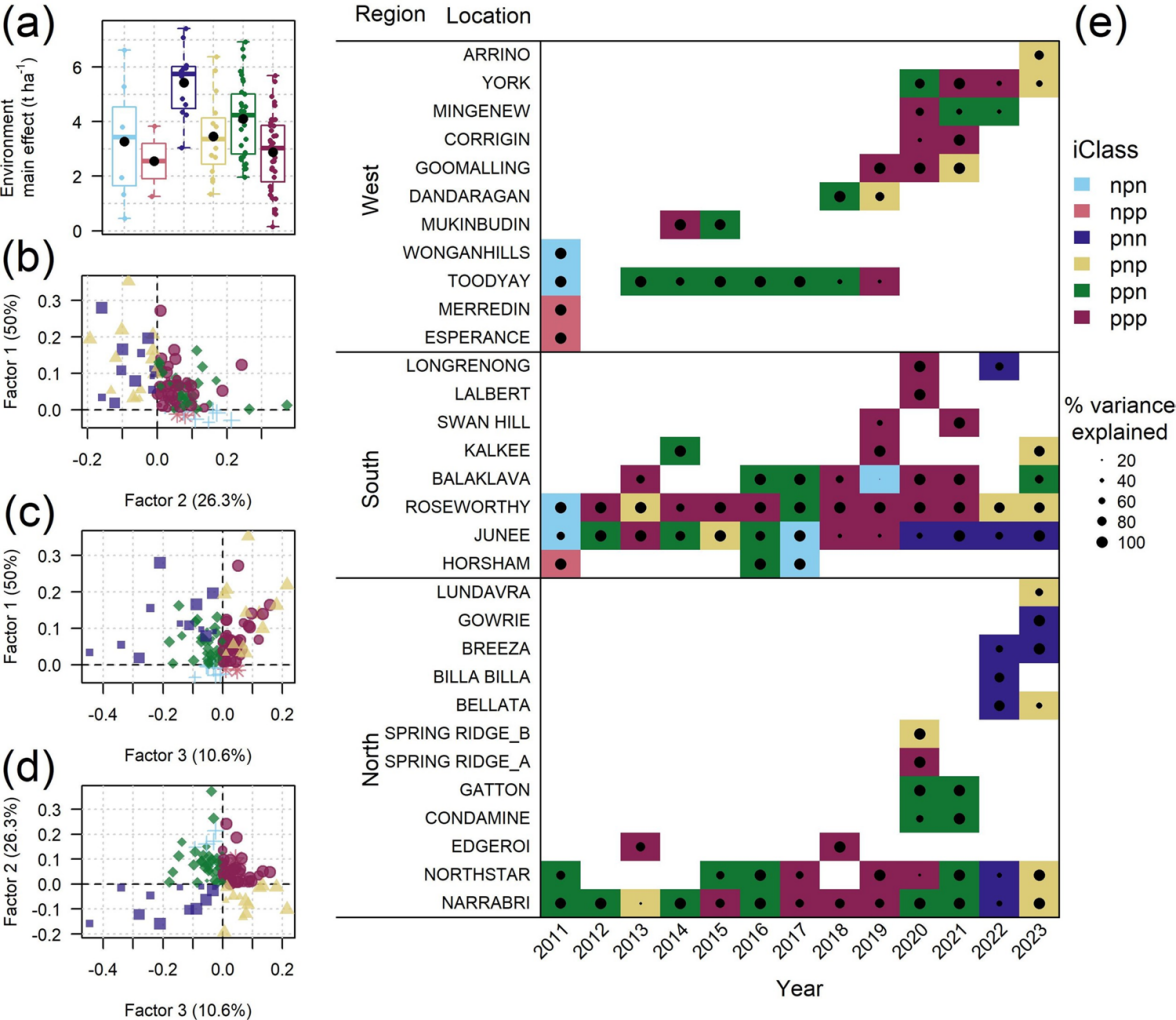
Distributions of yield main effects across iClasses (**a**); interaction classes (iClasses) are defined from positive and negative factor loadings (**b**–**d**); patterns of estimated iClass identity for 110 trial environments across regions, locations and growing years (**e**). The size of the points in plots b–d and within each cell in plot e indicates the percentage of the genetic variance explained by the FA3_GRM_ model for each trial environment

Comparison of iClasses in thirteen trial years across major growing regions revealed that iClasses were not specific to either regions, locations or years (Fig. 3). Nevertheless, some trends across regions within years were apparent. For example, in 2019, all environments in the North and South regions were assigned the ppp iClass, except for Balaklava where only 6.7% of the variance was explained, and three of the four environments in the West were also assigned the ppp iClass. The pnn iClass was also never assigned in the West in any year. Single locations rarely had consistent iClasses even when tested over many years. Junee in the North, which was included in all thirteen years, was allocated to every iClass except npp in at least one year. Narrabri in the North was described as iClass ppn for six of the thirteen years. In contrast, Roseworthy in the South was assigned to iClass ppn only once in 2017 and instead was iClass ppp for eight of the thirteen years. These patterns are illustrated in (Fig. 3).

### Environmental covariates characterise environment interaction classes

**Fig. 4:**
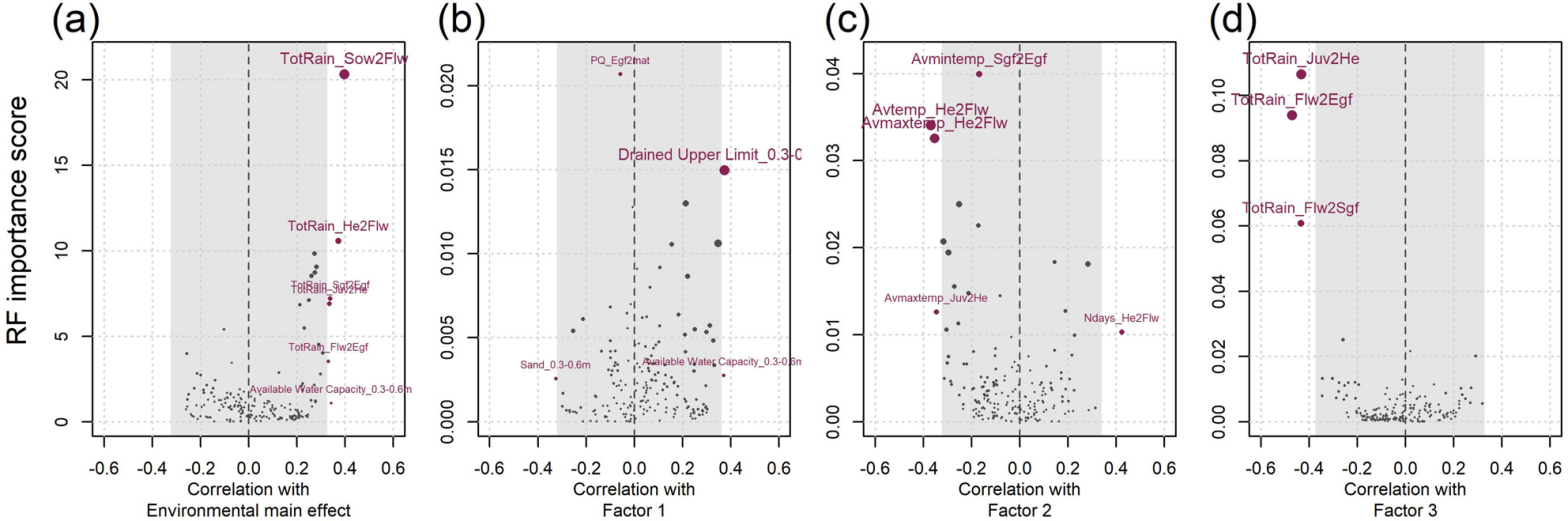
Correlation coefficients for each environmental covariate and factor analytic mixed model environmental main effects (**a**), factor loadings (**b**–**d**) Importance scores of each environmental covariate are derived from multivariate random forest prediction models. Grey areas indicate 95% confidence intervals based on one thousand permutations of random sampling

We found environmental main effects and factor loadings defined by FA models could be related to observed environmental covariates (ECs) to define generalised environment types of each iClass. Key associated ECs were identified by comparing correlations between ECs and both environmental main effects and factor loadings estimated from the FA3_*GRM*_ model in addition to importance scores derived from fitted random forest multivariate prediction models. Environmental main effects on yield were positively correlated with rainfall throughout the season where total rainfall between sowing and flowering, juvenile to heading, heading to flowering, flowering to end of grain fill, and start of grain fill to end of grain fill were all positively correlated (0.33 < r < 0.40) and were among the most important explanatory variables in random forest models (Fig. 4a). Some soil characteristics were also positively associated with environmental main effects on yield, including organic carbon at the 0.6 to 1 m soil depth and available phosphorus at the 0.15 to 3 m soil depth. The six environments that received more than 200 mm of rainfall between sowing and flowering and had more than 0.5% organic carbon at the 0.6 to 1 m soil depth all had high yield main effects between 5.55 and 6.94 t ha^−1^, while the eight environments that received less than 100 mm of rainfall between sowing and flowering and had less than 0.4% organic carbon at the 0.6 to 1 m soil depth had much lower yield main effects between 0.61 and 4.07 t ha^−1^.

The 29 ECs with the strongest positive or negative correlation with the first factor loading (0.25 < r < − 0.25) were soil property covariates from environments with strong positive first factor loadings (Fig. 4b). For example, Kalkee and Breeza (2023) and Spring Ridge (2020) soil was described by high drained upper limit, available water capacity and cation exchange capacity, with heavier soil types and a high percentage of clay or silt and low percentage of sand.

The second factor loading, which explained the largest proportion of cross-over GEI effects but was unrelated to environmental main effects, was more clearly associated with temperature and the duration of growth stage related ECs. Key ECs with both strong correlations and high importance scores included the average daily mean temperature and average daily maximum temperature between the heading and flowering growth stages (Fig. 4c). Environments with strong positive second factor loadings, such as Roseworthy in 2016 and 2019 as well as Balaklava in 2016, had generally cooler average temperatures (10.0 to 11.7 °C) and a long interval (21 to 25 days) between heading and flowering. In contrast, environments with strong negative second factor loadings, such as Breeza and Narrabri in 2023 and Bellata in 2022, were located in the Northern region, with greater average temperatures between heading and flowering (15.0 to 17.7 °C) and shorter duration between these two growth stages (14 to 15 days).

Similarly to environmental main effects on overall yield, the third factor loading was strongly related to ECs quantifying rainfall throughout the growing season. Total rainfall between flowering and the start of grain fill, between flowering and the end of grain fill and between the juvenile to heading stages all correlated negatively with the third factor loading (r < − 0.43) and had high importance scores (Fig. 4d). Environments with a strong negative third factor loading, such as Bellata, Breeza and Junee in 2022, had high rainfall between flowering and the end of grain fill (between 162.5 and 207.2 mm). Similarly, Junee and Horsham in 2016 had high rainfall between the juvenile and heading growth stages (between 174 and 289.7 mm). In contrast, environments with strong positive third factor loadings, such as Roseworthy (2013, 2015, 2020 and 2021), Narrabri (2019) and Spring Ridge (2020), received lower rainfall (between 0.5 and 61 mm) between flowering and the end of grain fill as well as the period between the juvenile and heading growth stages (between 13.4 and 130.7 mm). These environments could therefore characterised as relatively drought stressed environment types.

The environmental characteristics of all factor loadings could then be used to characterise environment types of each iClass group of environments. For example, the pnn iClass demonstrated the highest average yield main effects (5.42 t ha^−1^) and therefore more favourable environmental conditions. The pnn iClass associated factor loadings were defined by more fertile soils with better water holding capacity, which was indicated by the positive first factor loading. In addition, higher temperatures after heading associated with the negative second factor loadings and greater rainfall associated with the negative third factor loadings further described these favourable environments. In contrast, environments in the npp and ppp iClass had the lowest average yields (2.55 and 2.89 t ha^−1^, respectively) due to less favourable environmental factors, particularly including lower rainfall and more severe drought stress (indicated by positive third factor loadings). Despite these trends, definition of iClasses based on individual ECs was difficult with considerable overlap in ranges of ECs between iClasses.

### Prediction of environmental main effects and factor loadings

**Fig. 5:**
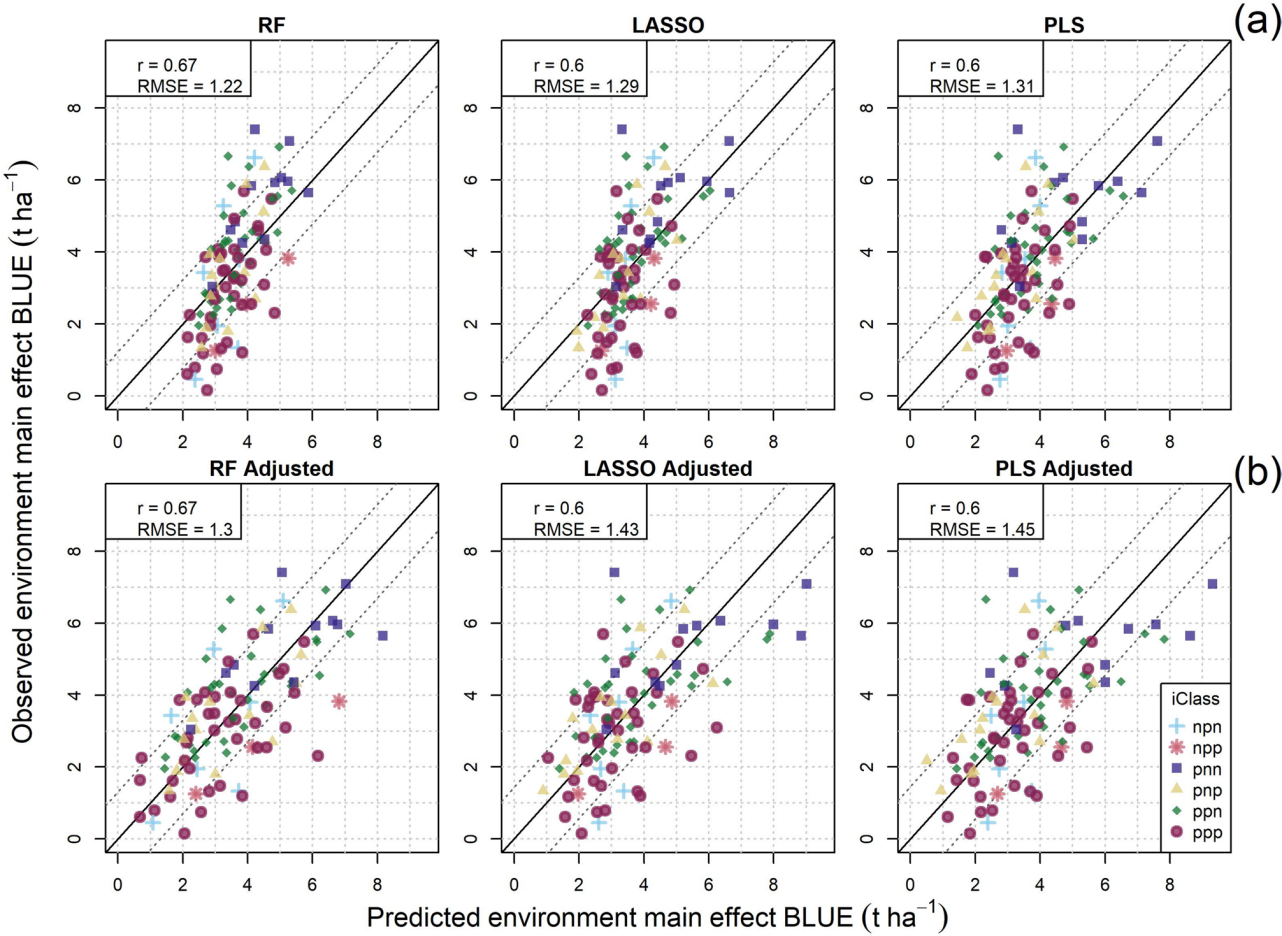
Model comparisons of observed and predicted (**a**) and adjusted predicted (**b**) values of environment main effects on yield. RF = random forest; LASSO = least absolute shrinkage and selection operator; and PLS = partial least squares. RMSE = root mean squared error and r = predictive ability. Predicted values were adjusted to minimise bias and shrinkage of predicted values. Solid diagonal lines indicate x = y, and diagonal dashed lines indicate RMSE thresholds

We tested several multivariate models for the prediction of environment main effects on yield and estimated factor loadings for test environments with known ECs in a leave-one-environment-out cross-validation strategy. Random forest models most accurately predicted environment main effects (r = 0.67) with the lowest error (RMSE = 1.23 t ha^−1^; Fig. 5). Noticeable shrinkage of predicted values was evident, particularly for random forest models, where the variance of the predictions (0.59) was much smaller than the variance of the observed environment main effects (2.31). Adjusted predictions with equal mean and variance as the observed environment main effects showed slight increase in error but similar differences among models (Fig. 5).

**Fig. 6:**
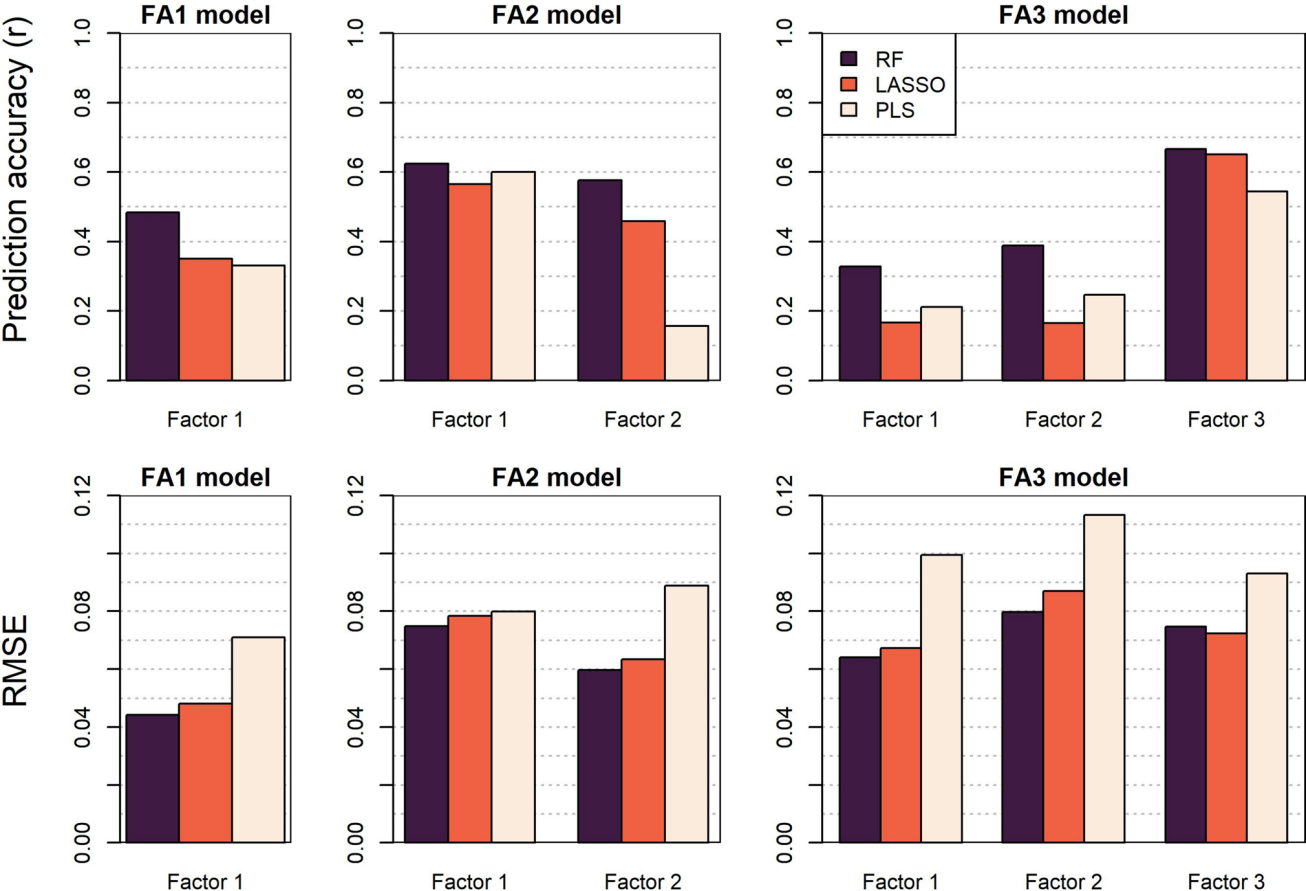
Comparison of predictive ability and root mean squared error (RMSE) of three multivariate prediction models for predicting factor analytic model factor loadings. RF = random forest; LASSO = least absolute shrinkage and selection operator; and PLS = partial least squares

For prediction of factor loadings influencing GE, random forest models performed best with the greatest predictive ability and the smallest RMSE. Interestingly, prediction accuracies were greater for higher order factor loadings in more complex FA3 models which explained only a small proportion of GE variation (Fig. 6). The sign (positive or negative) of the three factor loadings was correctly predicted for 90.9, 73.6 and 70.0% of environments, respectively, by a random forest model. Furthermore, iClass identities derived from the predicted factor loadings were correctly predicted for 44.6% of all environments. The two most frequent iClass environment types (ppn and ppp) had relatively high frequencies of 30.9 and 35.5%, respectively, while they were more frequently correctly predicted at 70.6 and 56.4% of the time, respectively. Conversely, models were unable to predict the rarer npn and npp iClasses that were never correctly predicted, indicating significant generalisation of this stage in environment type predictions. Importantly, the multivariate predictive ability of both environmental main effects and factor loadings were considerably greater than the strongest correlations with any single EC, suggesting that multiple soil and weather ECs defined over multiple crop growth intervals act in combination to influence grain yield and are essential parameters to predict GEI.

### Definition of Australian wheat mega-environments

**Fig. 7:**
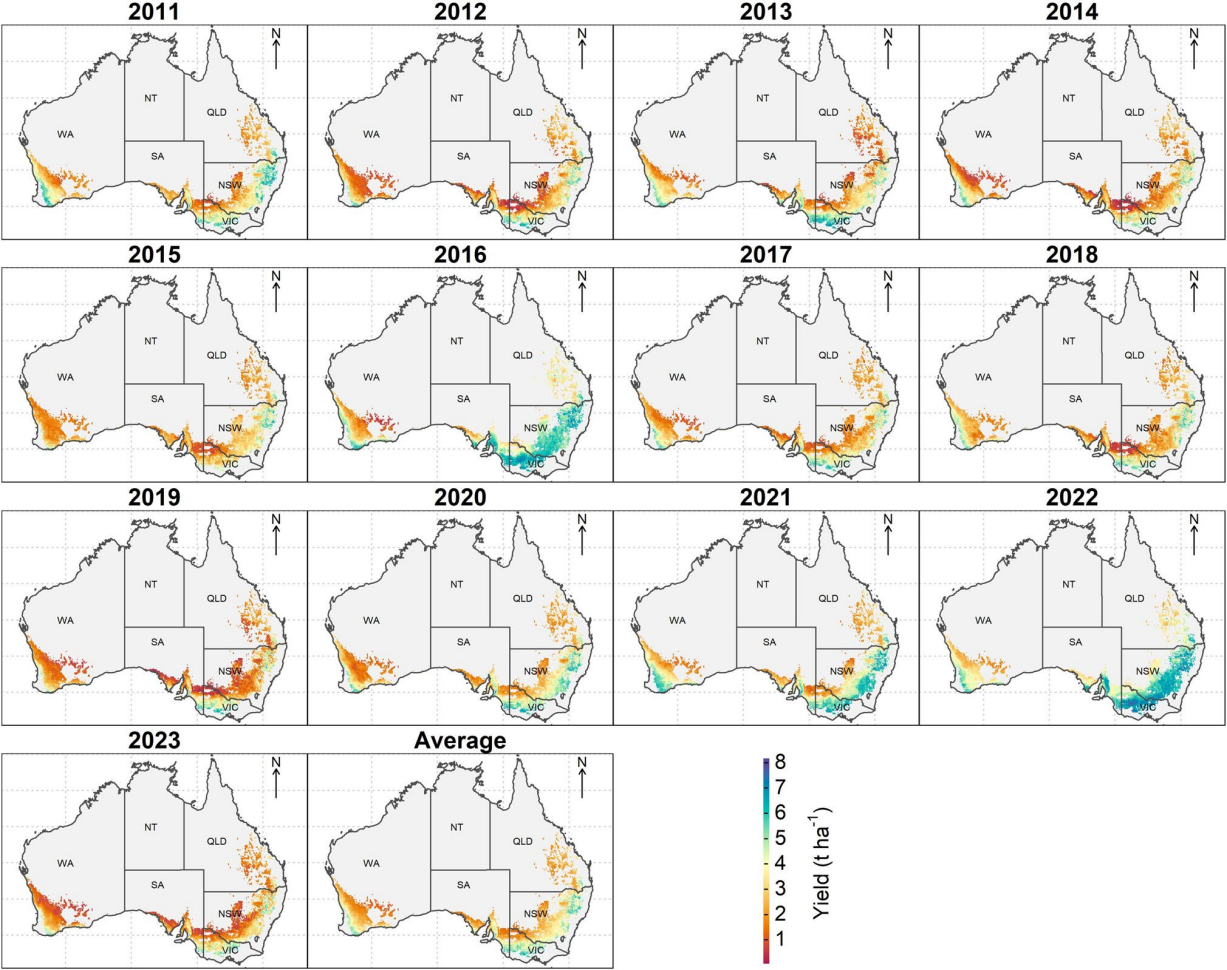
Maps illustrating predicted yield main effects across Australian wheat belt locations. Predictions were made from soil and weather environmental covariates from 2011 to 2023

After demonstrating that environmental main effects and factor loadings for untested environments can be predicted with reasonable accuracy, we used the full fitted FA3_GRM_ model to predict yield, GE factor loadings and iClass identity for 28,397 locations covering the Australian wheat belt for 13 years from 2011 to 2023. In total, this represented prediction of genotypes into 369,161 new environments. Maps of predicted mean yield each year clearly illustrated that higher rainfall regions closer to the coast in the Northern and Southern growing regions and the south-west of the Western growing region were associated with higher predicted yields (Fig. 7). Dramatic year-to-year variability was highlighted with the greatest predicted yields occurring in the years where actual rainfall was high (2016, 2022). The lowest prediction for yield was for 2019 and this year coincided with widespread drought in Australia (Wittwer and Waschik 2021).

**Fig. 8:**
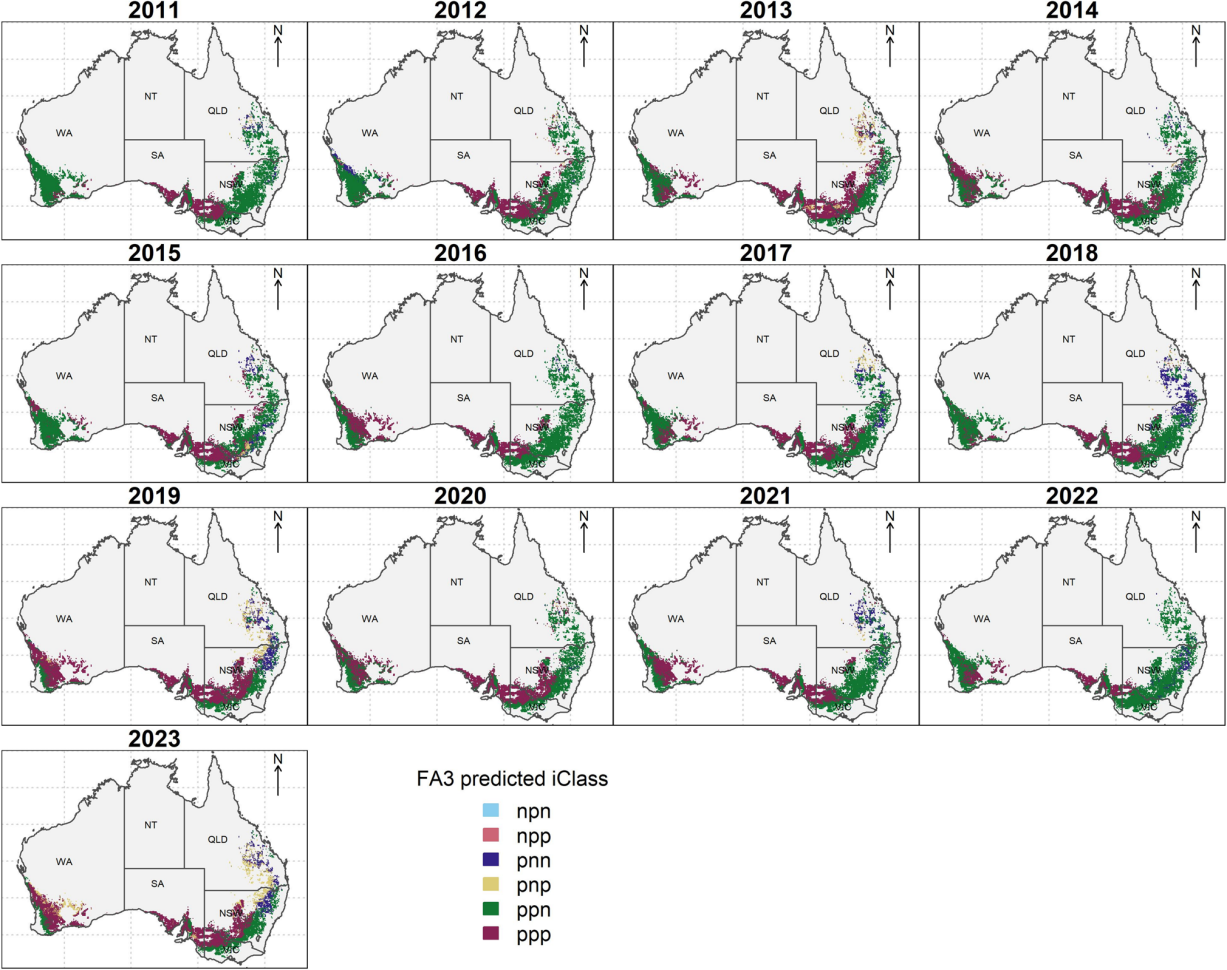
Maps illustrating predicted iClass identity across Australian wheat belt locations based on soil and weather environmental covariates from 2011 to 2023

Compared to the inconsistent trends in iClasses across the tested trial environments in the MET dataset (Fig. 3), mapping the predicted iClass identity over the landscape revealed more interpretable trends in iClass environment types within years, and shifts in distributions of iClasses due to year-to-year variability (Fig. 8). Patterns of iClass environment types were not separable at the scale and boundaries of major growing regions and often changed considerably at small scales within regions or between years. For example, when particularly low yields were predicted across most of the Western Australian region in the low rainfall year of 2019, a greater proportion of ppp iClass environments were predicted, whereas in the higher rainfall years of 2022 (all regions) and in the northern and southern regions (2016), the ppn iClass was more frequently predicted to occur.

**Fig. 9:**
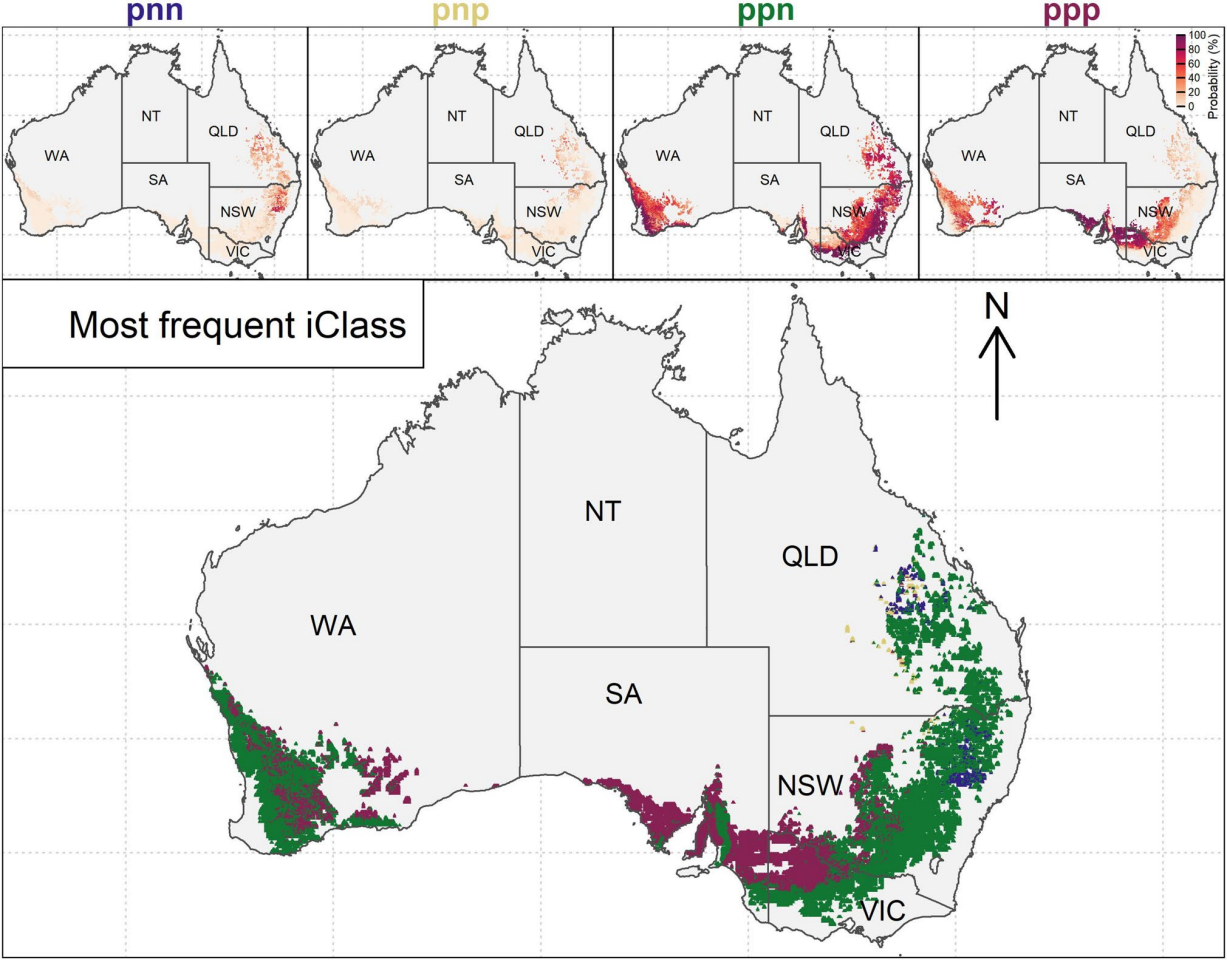
Maps of probability of predicted iClasses occurring between 2011 and 2023 across Australian wheat belt locations based on soil and weather environmental covariates. Upper plots show the predicted distributions of probabilities of each iClass occurring over the multiple years while the lower plot shows the most frequently predicted iClass at each location

Despite shifts in predicted iClass identity across regions due to seasonal year-to-year weather variability, considering the patterns of iClass frequency at each location across the period of 13 years revealed spatial distributions of mega-environments where each iClass would be more likely to occur considering static average climate conditions and soil properties (Fig. 9). The ppn iClass was the most frequently predicted across locations and was particularly prevalent closer to the coast in all growing regions that generally experienced higher rainfall. In contrast, the ppp iClass was more prevalent at drier inland locations (in particular, in the wheat growing areas of SA or the north-west of VIC) and was very rare in the northern region in QLD (Fig. 9). Overall, the pnn and pnp iClasses occurred rarely and only in the northern region in the north of NSW and QLD. Some locations, such as the south-west of the wheat Western growing region in WA, were highly likely to be associated with the ppn iClass, whereas locations such as the centre of the wheat belt in NSW or WA had much less certainty in the predicted iClass identity (Fig. 9).

### Genetic diversity adapted to Australian environments

**Fig. 10:**
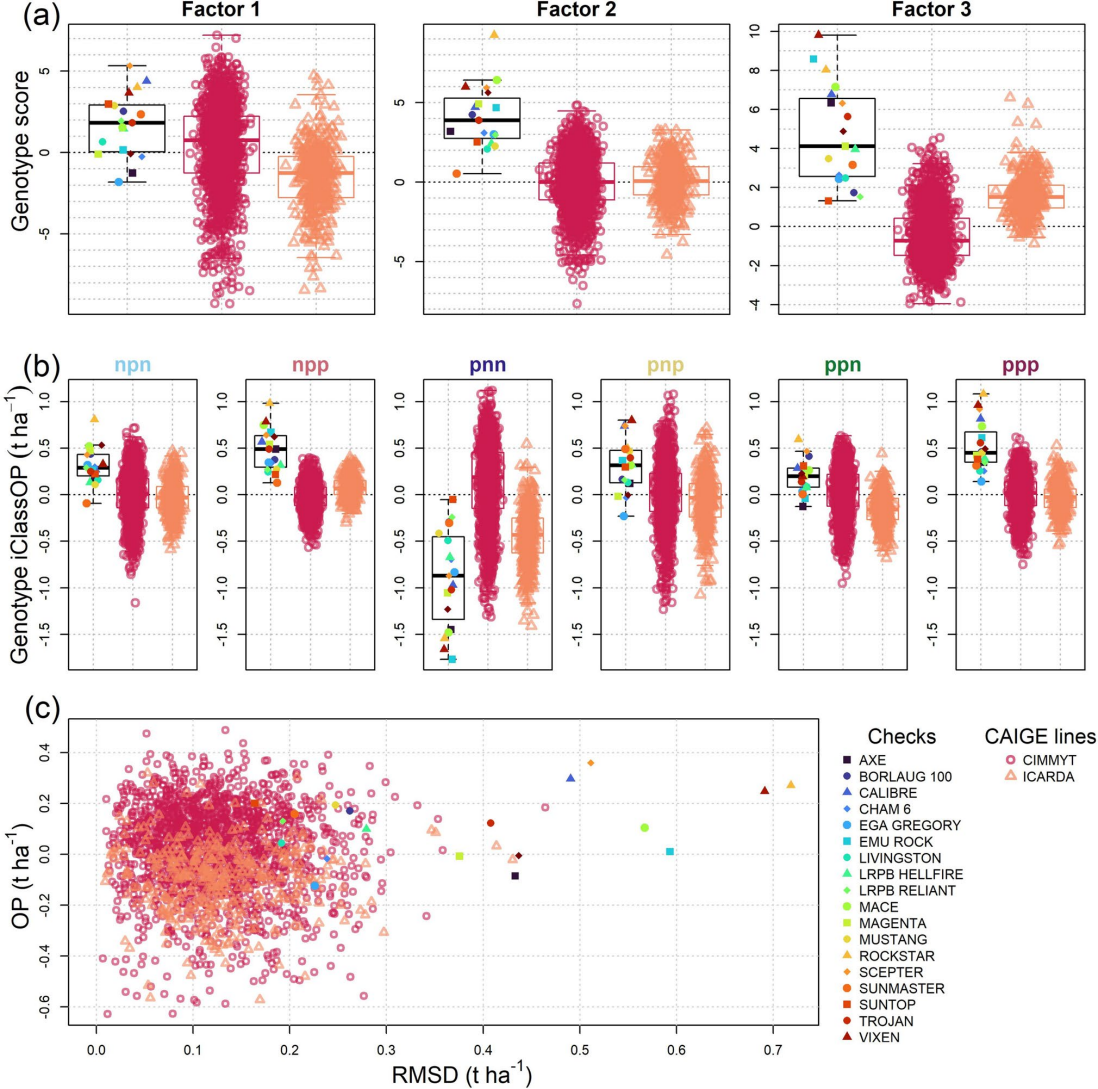
Comparisons of genotype adaptation for check varieties and CAIGE lines from the CIMMYT and ICARDA breeding programmes. Upper plots (**a**) show the genotype scores for each factor loading from the FA3_GRM_ model. Middle plots (**b**) show overall performance scores for each genotype for the average environment of each iClass (iClass OP). The lower plot (**c**) compares the overall performance across all iClasses (OP) over the first factor and yield stability over higher order factors (RMSD)

The different sets of genotypes used in this study to characterise GEI and iClasses included check varieties that are each well adapted to specific environment types that make up the wide range of Australian environments. Breeding lines from both the CIMMYT and ICARDA international wheat breeding programmes introduced through the CAIGE project were found to be more genetically diverse than the Australian check varieties (lower average genomic kinship estimates within groups) and were similarly distant between groups (Supplementary Fig. 2). The Australian check varieties Mace, Scepter, Rockstar, Vixen and Calibre are all highly connected through pedigrees and have closer kinship relationships than 99.89 and 99.78% of pairs of CIMMYT and ICARDA lines, respectively. These genetic resources may provide useful specifically or broadly adapted materials. Differences in genotype scores and predicted performance in each iClass environment illustrated where breeding has targeted specific environment types (Fig. 10). In general, the Australian check varieties had higher positive genotype scores for all three factors for the FA3_GRM_ model (Fig. 10a). This indicates (as expected) high overall performance across iClasses, but particular adaptation to cooler, drier and lower yielding iClasses environment types such as npp and particularly ppp (Fig. 10b), which both frequently occurred throughout all regions (Fig. 9). In contrast, ICARDA breeding lines demonstrated lower third factor loadings on average, and CIMMYT breeding lines demonstrated more negative third factor loadings (Fig. 10a), representing adaptation to warmer and wetter environments (pnn iClass) that was predicted to occur more often in the wheat growing regions of northern NSW and QLD (Fig. 10b). Although the Australian check varieties on average had the highest overall performance in the ppn iClass, there was a wide range in overall performance of CIMMYT lines for the ppn iClass. Several of these showed the greatest overall performance, highlighting the potential of this material for breeding in Australia (Fig. 10b). Also of note, there was particularly large variance in third factor genotype scores among Australian check varieties (Fig. 10a) and these also often had high RMSD across all iClasses (Fig. 10c). This highlighted cultivars, such as Rockstar and Vixen, that had high overall performing and high RMSD and so were particularly adapted to specific environment types. These two checks were ranked first and second, respectively, for iClass overall performance for both the npp and ppp iClasses but displayed the third and second lowest iClass overall performance in the pnn iClass (Fig. 10b). Conversely, varieties such as Sunmaster and Suntop demonstrated similar patterns of adaptation relative to the CIMMYT material, performing relatively poorly in npp and ppp iClasses but particularly well in the pnn iClass compared to other checks (Fig. 10b). Of note, the genotypes with the greatest overall performance and low RMSD (Fig. 10c) were often CIMMYT derived CAIGE lines, underlining their strong potential for broad adaptability and yield stability.

To further investigate mechanisms of genotype adaptation and GEI, we compared how genotype scores for each factor relate to phenology and plant height traits within each group of genotypes. Among all comparisons, only a weak but significant correlation was found between genetic effects for estimated days to heading and the third factor score for CIMMYT lines (r = 0.16, *p* < 0.001). This suggests that earlier maturity CIMMYT lines demonstrated lower third factor scores and greater overall performance in pnn and ppn iClass environments. Associations were also found for plant height where a small but significant negative correlation with the third factor scores was found for CIMMYT lines (r = − 0.15, *p* < 0.001), but this association was much stronger for the Australian checks (r = − 0.89, *p* < 0.001). This suggests that shorter varieties and genotypes within this height range from both groups had generally higher third factor scores and greater performance in the more drought stressed pnp and ppp iClass environments. This trend was also reflected by differences in reduced height gene status among lines where semi-dwarf *Rht-1* (*Rht-B1b* and *Rht-D1a*) genotypes had a significantly (*p* < 0.001) lower mean third factor genotype score of 1.2 compared to a mean of 3.0 for *Rht-2* (*Rht-B1a* and *Rht-D1b*) genotypes and concurs with results from Eagles et al. (2014). Approximately 96 and 95% of lines tested from the CIMMYT and ICARDA breeding programmes, respectively, were semi-dwarf *Rht-1*.

### Genomic prediction into untested environments

**Fig. 11:**
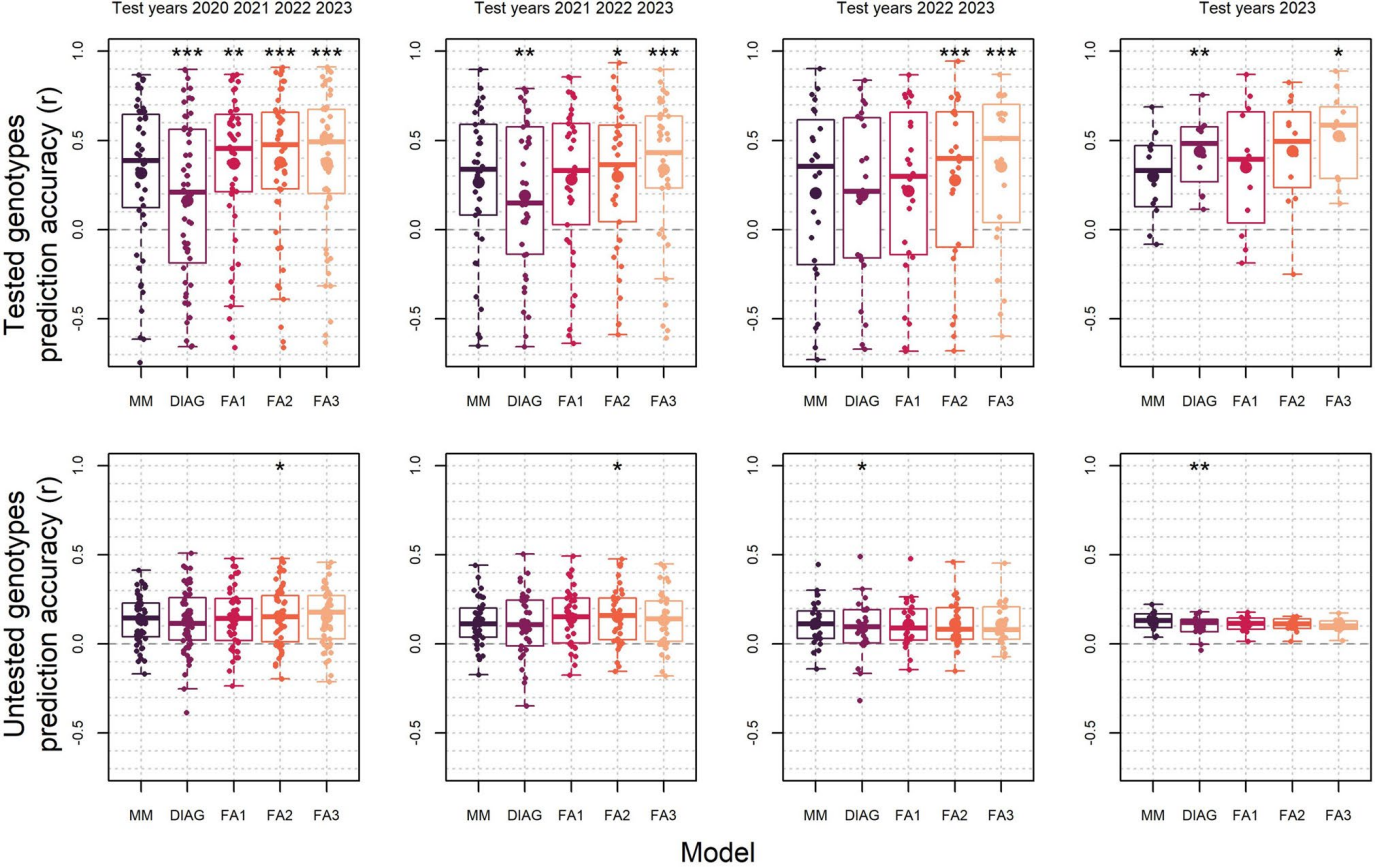
Boxplots comparing within trial environment predictive ability of different models over four forward prediction cross-validation schemes. Each point indicates a predictive ability for a single environment, and large points indicate the mean predictive ability or each model. MM is the genetic main effects models, DIAG is diagonal genetic covariance structured models, and FA1 to FA3 are factor analytic structured models. Asterisks above each boxplot indicate levels of significant differences between each model and the MM baseline model from paired two-tailed t-tests where ‘*’ indicates *p* < 0.05, ‘**’ indicates *p* < 0.01 and ‘***’ indicates *p* < 0.001

We further validated the above approach of prediction of factor analytic mixed model factor loadings with observed ECs by testing a forward genomic prediction cross-validation scheme into untested environments with known ECs. Predictive ability for check varieties that were tested over several years was significantly increased for higher order FA models compared to the baseline genetic main effects (MM) model in all cross-validation folds. This increase was smallest in the first cross-validation fold, which included 9 years of trial data in the training set, where prediction accuracies increased from 0.32 for the MM model to 0.38 for the FA3 model. When more years of data were used in the training set for the cross-validation fold including only 2023 as the test year, a greater increase in predictive ability was observed from 0.29 for the MM model to 0.53 for the FA3 model. (Fig. 11).

The increase in prediction accuracies was much lower for untested genotypes (CAIGE lines tested in single years). In most cases, there was no significant difference in predictive ability between DIAG or FA models compared to the MM baseline model. Nevertheless, prediction accuracies were slightly, but statistically significantly, increased from 0.14 and 0.13 for MM models to 0.16 and 0.15 for FA2 models in the 2020 to 2023 and 2021 to 2023 cross-validation test folds, respectively (Fig. 11). Overall, these results confirm that prediction of factor loadings with observed ECs facilitates increased prediction of GE effects when genotype scores are estimated from tested genotypes, or from accurate genomic predictions.

## Discussion

Wheat breeding in Australia is hindered by prevalent and unpredictable GEI. By making use of a large dataset of MET series and well-established factor analytic mixed model analysis methods, we demonstrated that environmental main effects on yield and latent factor loadings that drive GEI can be related to and are predictable from observed ECs. Taking a similar approach to Araújo et al. (2024) and Costa-Neto et al. (2020), second-stage models to predict these parameters were used to predict out to a much larger expanded set of environments that cover Australian wheat growing environments at a much broader scale across locations and time. Importantly, this mapping approach revealed generalised spatial and temporal trends in predicted GEI that were unclear when only considering the observed MET environments. Yield main effects and environment types were both significantly impacted by year-to-year variability which emphasises the importance of multi-year, multi-environment testing to select broadly adaptable genotypes to these non-static environmental effects. Nevertheless, by predicting into a large number of environments that thoroughly represent the Australian wheat TPE over several years and considering the probability of predicted iClass environment types occurring at each location over several years, some trends in static GEI effects could be used to define mega-environments of locations for which breeding can target specifically adapted genotypes (Basford and Cooper 1998). Importantly, these results justify considering each year-location environment uniquely independent rather than modelling separate location and years random effects and demonstrates how considering predictions for a long-term distribution of weather scenarios based on historical observed weather enables differentiation of static and non-static GEI effects that would be likely to occur in future years. In practice, selection for high overall performance in one iClass would be the best approach for locations with a high probability of that iClass occurring. However, if multiple iClasses are predicted to occur with reasonable probability over several years, then a more complex approach of selection for high overall performance proportional to the multiple iClasses in combination with low RMSD stability values would be required.

Heslot et al. (2014) considered the analogy of the Anna Karenina effect; that ‘Happy families are all alike; every unhappy family is unhappy in its own way’, for the unpredictability and difficulty in generalisation of GEI due the large number of independent environmental effects that may limit yield. We found that common GE effects variance was much larger than that for specific effects. This may be due to the large number of environments and seasons that the MET effectively sampled from the target population of environments (TPE) covering the diverse Australian wheat grain belt. Thus, contrary to the Anna Karenina effect, we found that generalisation of GEI and predictability into untested environments is possible to some extent. Nevertheless, we acknowledge that methods presented here inherently generalise GEI, first through factor analytic approximation of common rather than environment-specific genetic effects considered random rather than fixed, and secondly through predictions in the second-stage model including ECs out to the wider set of environments with another degree of error and uncertainty. As a result, finer scale and rare GEI may well be overlooked. We observed significant shrinkage towards the mean of predictions of environmental main effects. Despite attempts to rescale the variance of predictions through cross-validation, predictions may be less accurate in extreme environments. We also note that generalisation of GEI is inherent to the scale of the TPE; in that similar approaches using MET data for a more refined TPE would likely resolve finer scale GEI effects and mega-environments better than presented here. The genotypes used in this study also likely influenced the generalised environment type effects. We found that the CAIGE wheat lines from both CIMMYT and ICARDA represent a more diverse and distant genepool than the Australian check varieties and demonstrated novel adaptation patterns across environment types. Different results, in terms of iClass mega-environment trends and boundaries, would likely be found using a different panel of genotypes or lines within specific breeding programmes with different selection histories. We also anecdotally observed that the Australian checks often displayed contrasting genetic correlations compared to CAIGE lines. Further work may investigate whether fitting different covariance structures to groups of related genotypes increases predictive performance.

The iClass environment types that more frequently occurred in northern NSW and QLD were identified as target environments for CAIGE material (particularly from CIMMYT) adaptation. These findings agree with previous work that found high genetic correlations between environments in the northern Australian wheat growing region and the CIMMYT selection environments in Obregon, Mexico and that this is likely due to the similar temperature and period of the growing season as well as the deep soil profile (Cooper et al. 1993; Cooper and Woodruff 1993; Mathews et al. 2007). Historical use of CIMMYT material, particularly in Australian breeding programmes in NSW and QLD, also supports these trends (Brennan and Quade 2006). We also note that the combination of high overall performance and low RMSD of many CIMMYT lines in CAIGE trials compared to specifically adapted checks highlights the value of these broadly adaptable materials across diverse Australian growing environments.

We found that factor loadings from a first stage MET model could be associated with ECs and were predictable with moderate predictive ability from second-stage multivariate models using many ECs. The two-stage modelling approach enabled a large and complex MET dataset to be fitted in the first stage. Fitting one-stage MET analysis models including ECs with latent or factorial regression structures, as applied by Tolhurst et al. (2022) or Piepho and Blancon (2023), may be a more direct approach and could enhance the predictive ability and generalisability of models, but increases the number of parameters to be estimated in a mixed model. In contrast, we found that the random forest machine learning approach in the second-stage model including ECs was highly computationally efficient and flexible to modelling complex interactions among ECs without the need for restrictive assumptions of variance structures. Standard methods to integrate ECs into mixed model frameworks would also only model linear additive effects. While multivariate models provided better predictive ability than single EC associations, inference of direct causal effects from any single EC is problematic due to the high collinearity and potentially confounding effects among ECs. For example, the strong north to south temperature gradient is confounded with day length, and soil constraints in South and Western Australia are also known to be more severe than in the northern growing regions of NSW and QLD, which have summer rather than winter dominated rainfall patterns. Nevertheless, combined modelling of ECs is pragmatic considering non-additive effects among ECs are expected. For example, multiplicatively more severe drought stress might be expected due to low rainfall at locations with lighter soils with poorer water holding capacity, and patterns of soil characteristics and the rainfall gradient across the Australian grain belt are largely independent. The better performance of random forest prediction models, which can model interaction effects among predictors, also suggests that non-additive effects among ECs are important. Further research to validate and refine the GEI effects may involve trial experiments to control particular environmental effects, such as heat or drought stress, with more directed populations of genotypes for quantitative trait loci mapping, similarly to Bennett et al. (2012), or with near-isogenic lines that contrast for key known genetic effects, similarly to Chapman et al. (2007).

An advantage of our approach is that it builds upon a well-established framework of MET analysis with factor analytic linear mixed models that many crop breeders routinely apply in large commercial breeding programmes (Smith et al. 2021). While analysis of large METs is often limited by computational capacity and software for fitting complex linear mixed model covariance structures (Smith et al. 2023), further work could better integrate ECs into single-stage MET analyses or build assumptions of covariance structures, that are of particular relevance to plant breeding contexts, into computationally efficient machine learning approaches. Definition of ECs from weather and soil data makes many assumptions of environmental effects on crop growth, and these methods could be further developed integrating insights from crop models and plant physiology. Standardised methods to process environmental data from multiple sources and define ECs for Australian growing environments will be facilitated by the EC4MET R package developed as part of this work.

In the present study, we used ECs that defined abiotic environmental stresses using weather and soil characteristics. Further work may integrate additional covariates that quantify biotic stresses  to further explain components of GEI. Several soil-borne pathogens are of particular importance across Australian wheat growing environments and can be quantified using DNA-based tests from soil samples (Ophel-Keller et al. 2008), and Thompson et al. (2021) found strong correlations between FA loadings and root-lesion nematode (*Pratylenchus thornei*) abundance in soil profiles in a wheat MET. Although many of the soil-borne pathogen species, such as crown rot (*Fusarium pseudograminearum*), are known to occur in defined regions (Alahmad et al. 2018), inference of such biotic ECs into many new and unobserved environments would be difficult compared to the well interpolated weather and soil datasets used here. Foliar pathogen incidence, such as stripe rust (*Puccinia striiformis* f.sp. *tritici*), is known to vary greatly due to year-to-year due to weather (Park 1990) and with incursions of new pathotypes (Wellings 2007). The static and non-static effects of abiotic ECs as demonstrated here, as well as more difficult to predict and quantify biotic effects on GEI, which may be influenced by management practices should also be considered pragmatically by plant breeders looking to define target regions.

Crop breeding programmes are subject to inertia; in that breeding cycles operate over long timespans and changes to breeding strategies or direction are slow to come to fruition. Breeders should therefore consider prevailing environment types and climates several decades into the future. Methods presented here to predict environment types into many new environments could well be applied using climate change projections to anticipate near-term impacts of climate change and ensure that genetic gain is prioritised and achieved in environment types that are likely to become more prevalent. Current climate projections indicate a high certainty of increased temperatures across Australia but projected changes in rainfall patterns are less consistent across independent climate models (Grose et al. 2020). Therefore, the higher average temperature iClass environment types identified here that were predicted to occur most frequently in the northern growing regions (iClasses pnn and pnp) may be considered of high importance in future climate change scenarios and highlights the potential value of novel CAIGE genotypes with high overall performance in these environments. These methods could also be applied to plant breeding for other crop species or for improved informatics for farmers’ variety selection using large variety testing trial networks where the farmer’s target environment is a season that has not occurred yet and at a location for which there have been no trial experiments.

## Conclusion

Insights from this work demonstrate how high-resolution environmental data can be processed and used by plant breeders to gain a better understanding of GEI effects to improve selection decisions. The value of specific adaptation indicated by static GEI effects, compared to broad adaptation influenced by non-static year-to-year GEI effects, will be important to consider. Finally, the increasing importance of climate change will make these approaches particularly relevant to modern crop breeding.

## Supplementary Information

Below is the link to the electronic supplementary material.

Supplementary file1 (XLSX 28 KB)

Supplementary file2 (PDF 329 KB)

## Data Availability

MET yield data are available through CAIGE project website (www.caigeproject.org.au). Some of the genotype data used in this study are available at (10.25919/3x88-4p26; Fradgley 2025a. Weather data are available through the SILO website (www.longpaddock.qld.gov.au/silo/), and soil data are available through the SLGA website (https://esoil.io/TERNLandscapes/Public/Pages/SLGA/index.html). The EC4MET R package (10.5281/zenodo.15400245; Fradgley and Whan 2025) has been developed to facilitate workflows to define ECs from weather and soil data for integration into MET analyses. Example analysis R scripts for fitting MET models and other functions described in this manuscript are provided (10.5281/zenodo.15400263; Fradgley 2025b).
