# Supplementary material for: Prediction of Australian wheat genotype by environment interactions and mega-environments
Source: Theor Appl Genet. 2025 Sep 4;138(9):241. doi: 10.1007/s00122-025-05023-6 (PMC12411322; doi:10.1007/s00122-025-05023-6)
Supplement: Supplementary file 2 — Supplementary file2 (PDF 329 KB) [file 122_2025_5023_MOESM2_ESM.pdf]

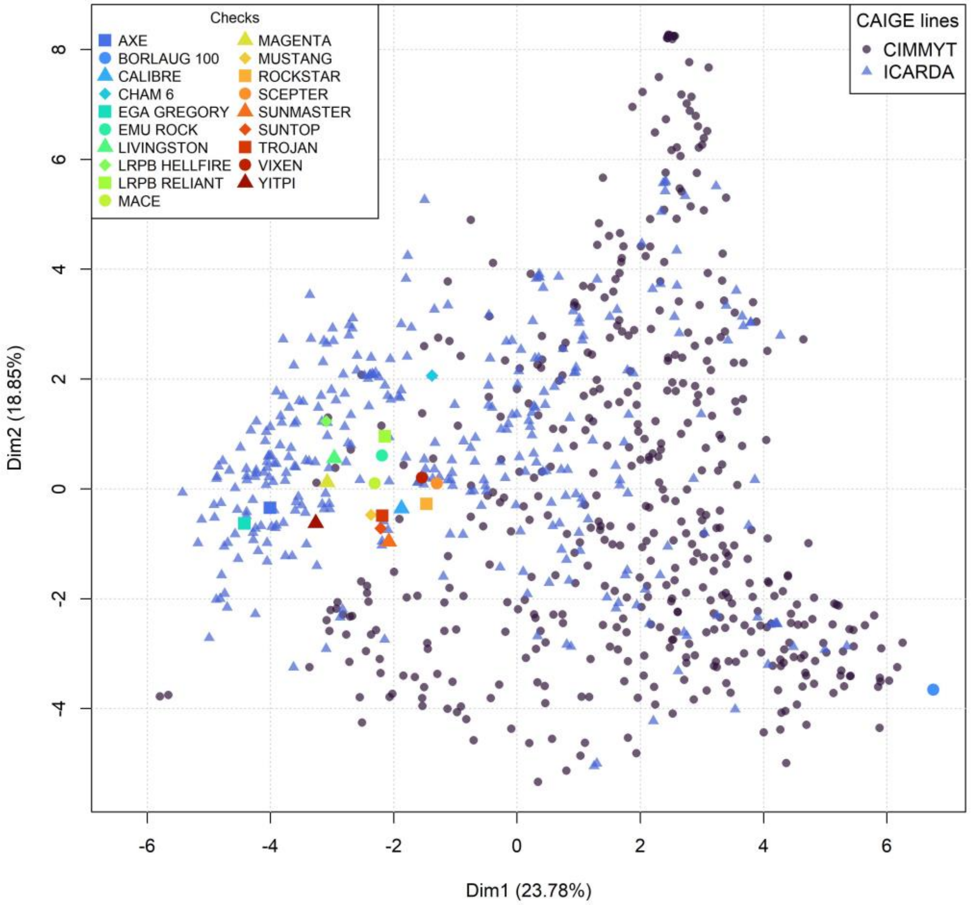

Supplementary Figure S1. Principal coordinate plot of the first two dimensions for genetic relationships among 824 CAIGE wheat lines and check varieties.

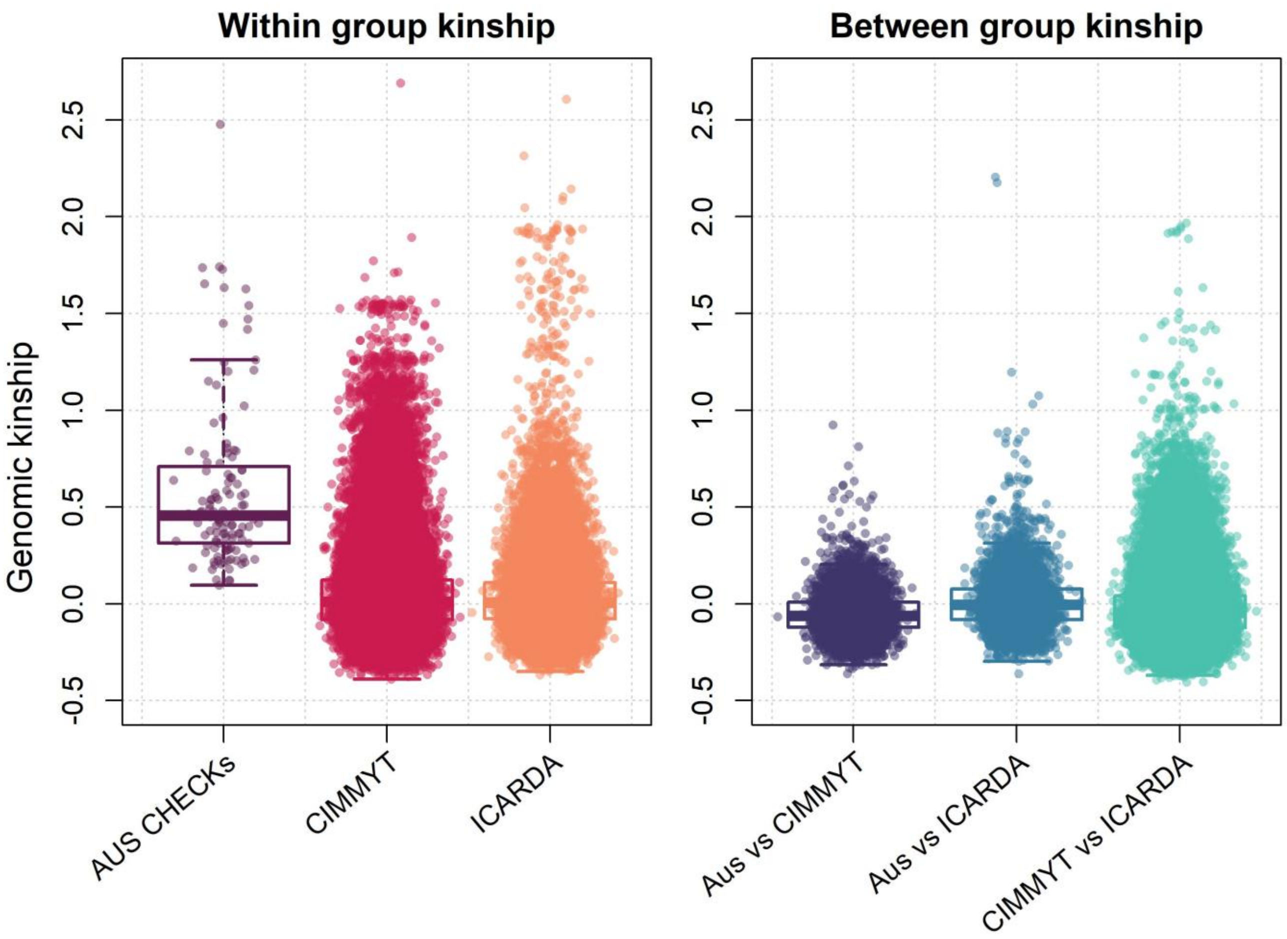

Supplementary Figure S2. Comparison of within and between group pairwise genomic kinship coefficients for Australian check varieties CAIGE lines from the CIMMYT and ICARDA breeding programs.
